# Supplementary material for: Biological relevance of computationally predicted pathogenicity of noncoding variants
Source: Nat Commun. 2019 Jan 18;10:330. doi: 10.1038/s41467-018-08270-y (PMC6338804; doi:10.1038/s41467-018-08270-y)
Supplement: Supplementary file 1 — Supplementary Information [file 41467_2018_8270_MOESM1_ESM.pdf]

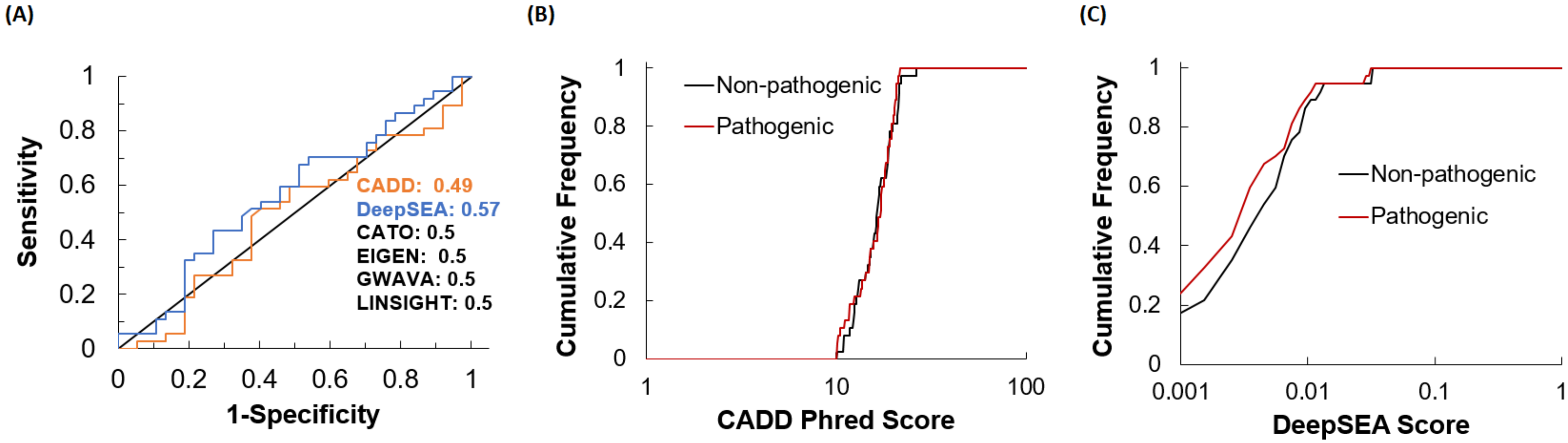

Supplementary Figure 1. Additional evaluation of diagnosing position-matched ncSNVs. A total of 37 pairs of ncSNVs at highly conserved positions were analyzed. (**A**) ROC curves with AUROC values displayed for each method. (**B**) Cumulative distribution of CADD scores. (**C**) Cumulative distribution of DeepSEA scores.

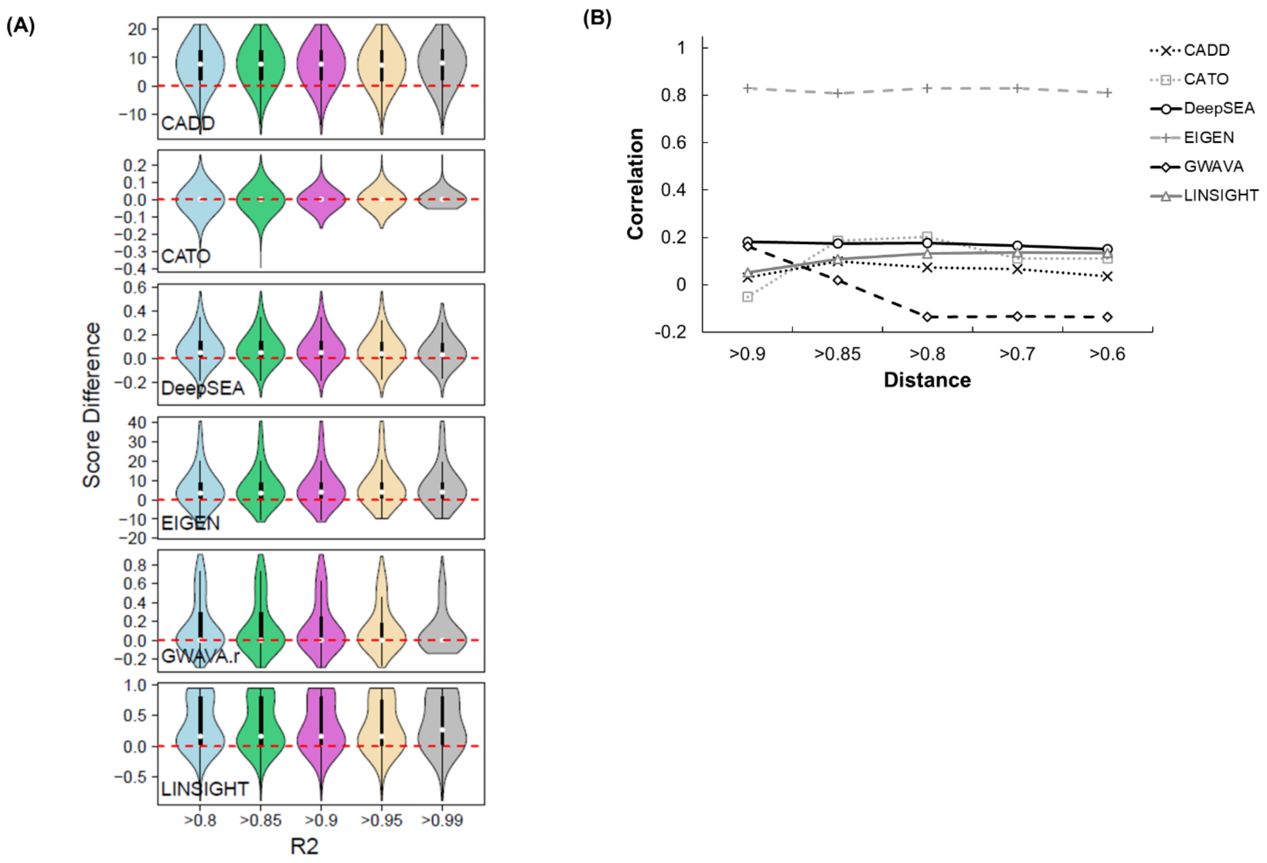

Supplementary Figure 2. Effect of linkage disequilibrium (LD) on discrimination of pathogenic ncSNVs. (**A**) Violin plots show distributions of impact score difference between nearby pathogenic and non-pathogenic ncSNVsfor each of the tested methods. Variants were grouped into bins based on LD blocks using R^2 values released by the 1000 Genomes Project. (**B**) Correlation of impact scores for ncSNVs located within given LD blocks. Pearson correlation coefficient values are displayed.

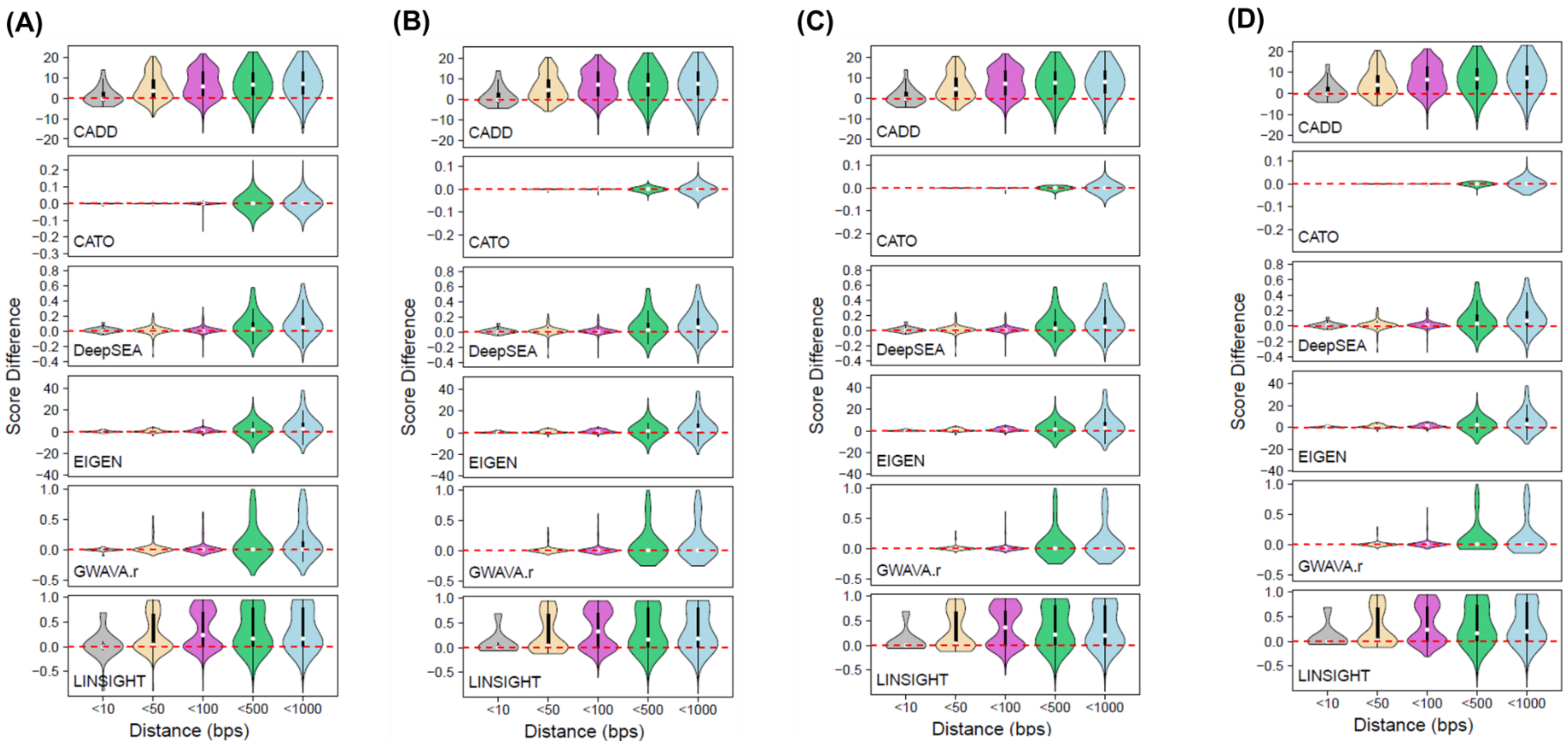

Supplementary Figure 3. Distributions of impact score difference between nearby pathogenic and non-pathogenic ncSNVs. In addition to the three filters applied to HGMD variants, we further required that pathogenic variants had population frequencies <1% (**A**), <0.1% (**B**), <0.01% (**C**) and 0% (**D**) in the GnomAD database.

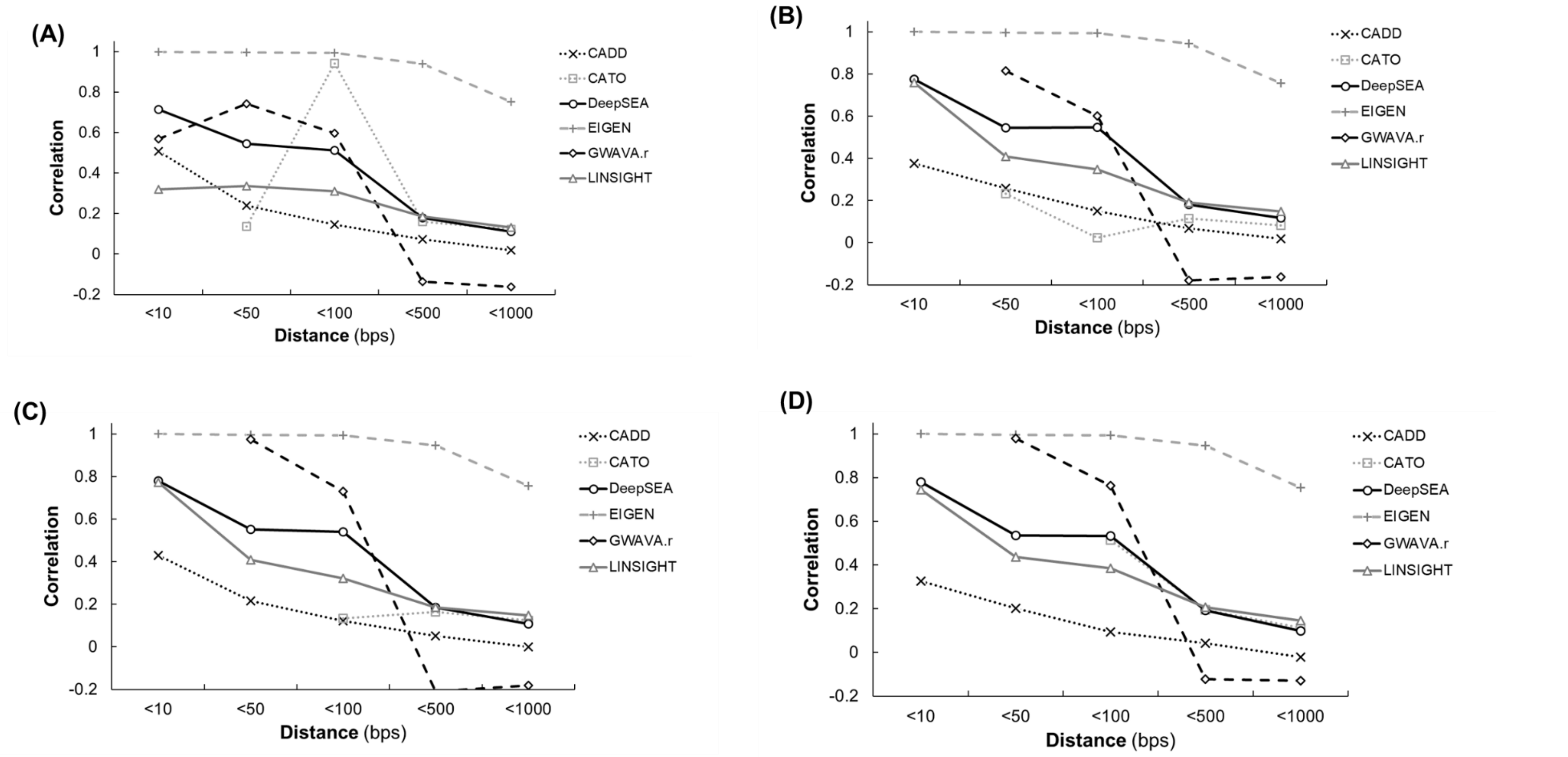

Supplementary Figure 4. Correlation of impact scores for ncSNVs located within given genomic distances. In addition to the three filters applied to HGMD variants, we further required that pathogenic variants had population frequencies <1% (**A**), <0.1% (**B**), <0.01% (**C**) and 0% (**D**) in the GnomAD database.

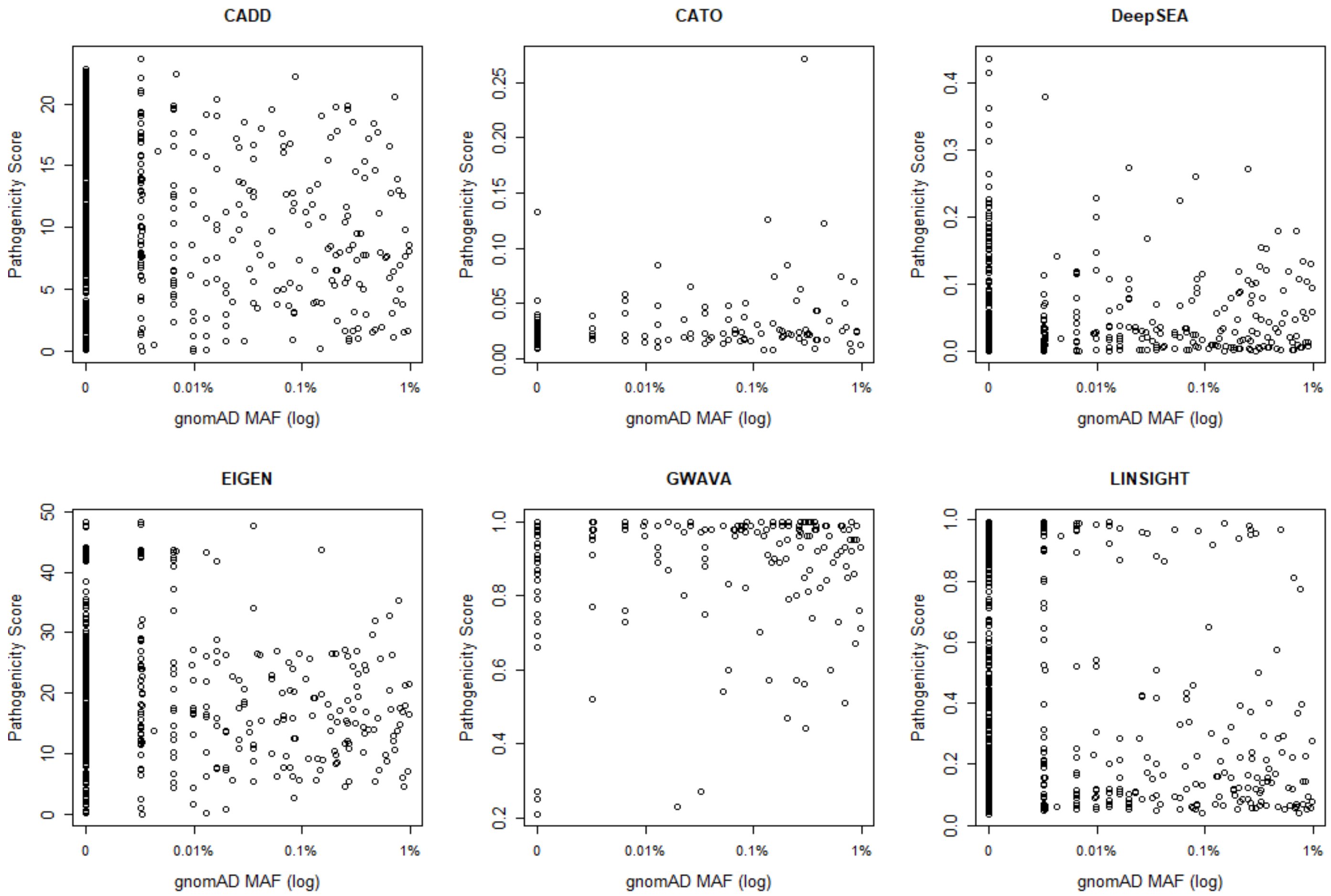

Supplementary Figure 5. Scatter plots showing the relationship between GnomAD population frequencies of pathogenic variants and impact scores produced by six methods.

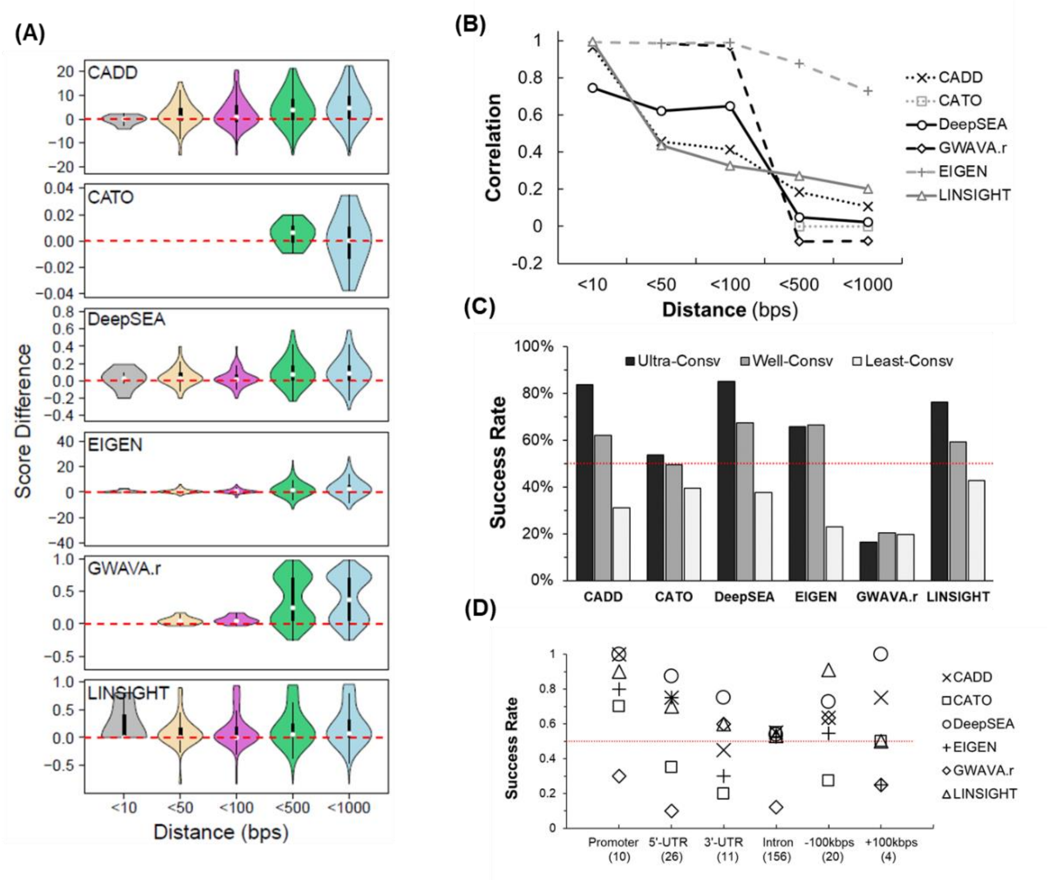

Supplementary Figure 6. Performance of tested methods on diagnosing ClinVar region-matched ncSNVs. DAVs were retrieved from the ClinVar database. (**A**) Violin plots show distributions of impact score difference between nearby pathogenic and non-pathogenic ncSNVs. Variants were grouped into bins based on distances measured by base pairs. (**B**) Correlation of impact scores for ncSNVs located within given genomic distances. (**C**) Success rate of ranking pathogenic ncSNVs to the top among non-pathogenic ncSNVs located within its 1,000 bps flanking region. Data are stratified by conservation of the position harboring pathogenic ncSNVs. (**D**) Success rate stratified by the genomic context of pathogenic ncSNVs, including promoters, 5'-and 3'-untranslated regions (UTRs), introns, near-gene (+/-100 kbps) regions and gene-desert regions.

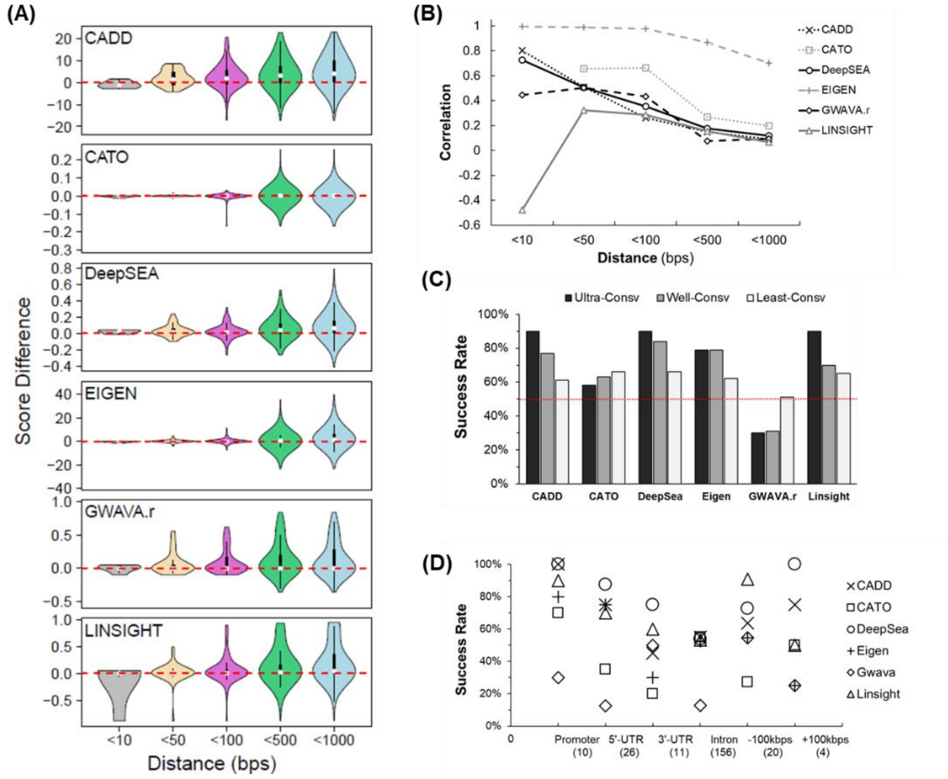

Supplementary Figure 7. Performance of tested methods on diagnosing unique region-matched ncSNVs. DAVs were retrieved from the HGMD database with three filters, but only one DAV per gene was used in the analysis. (**A**) Violin plots show distributions of impact score difference between nearby pathogenic and non-pathogenic ncSNVs. Variants were grouped into bins based on distances measured by base pairs. (**B**) Correlation of impact scores for ncSNVs located within given genomic distances. (**C**) Success rate of ranking pathogenic ncSNVsto the top among non-pathogenic ncSNVs located within its 1,000 bps flanking region. Data are stratified by conservation of the position harboring pathogenic ncSNVs. (**D**) Success rate stratified by the genomic context of pathogenic ncSNVs, including promoters, 5’-and 3’-untranslated regions (UTRs), introns, near-gene (+/-100 kbps) regions and gene-desert regions.

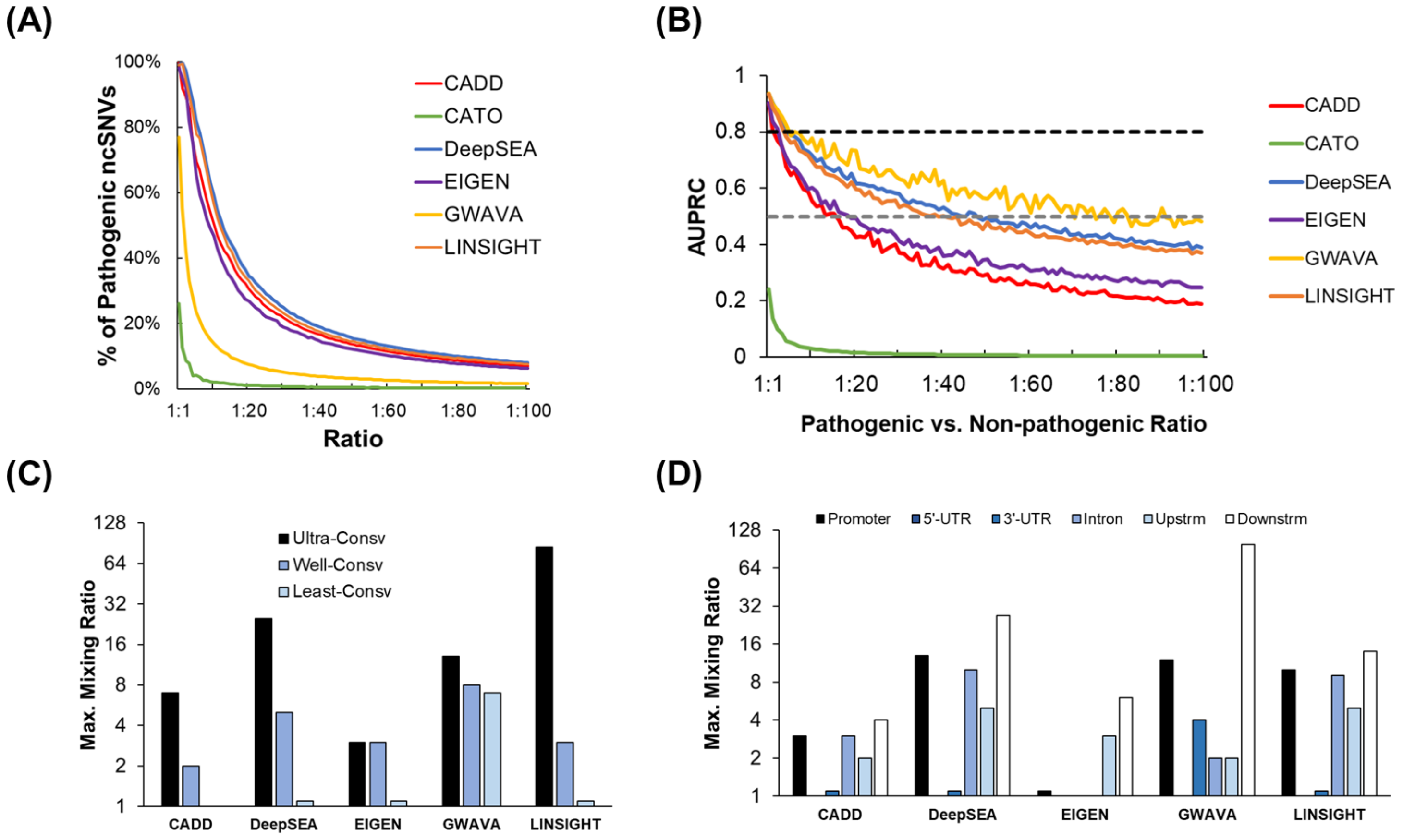

Supplementary Figure 8. Performance of six methods on prioritizing HGMD variants further filtered with population frequency < 0.01% in the GnomAD database. (**A**) Fraction of pathogenic ncSNVs in the top 10% percentile of impact scores declines exponentially as the mixing ratio increases. (**B**) AUPRC value decreases as the mixing ratio increases. (**C**) Maximum mixing ratio for each tool to achieve AUPRC > 0.8 when pathogenic ncSNVs disrupt ultra-, less-or least-conserved positions. (**D**) Maximum mixing ratio for each tool to achieve AUPRC > 0.8 when pathogenic ncSNVs is inside different types of genomic regions.

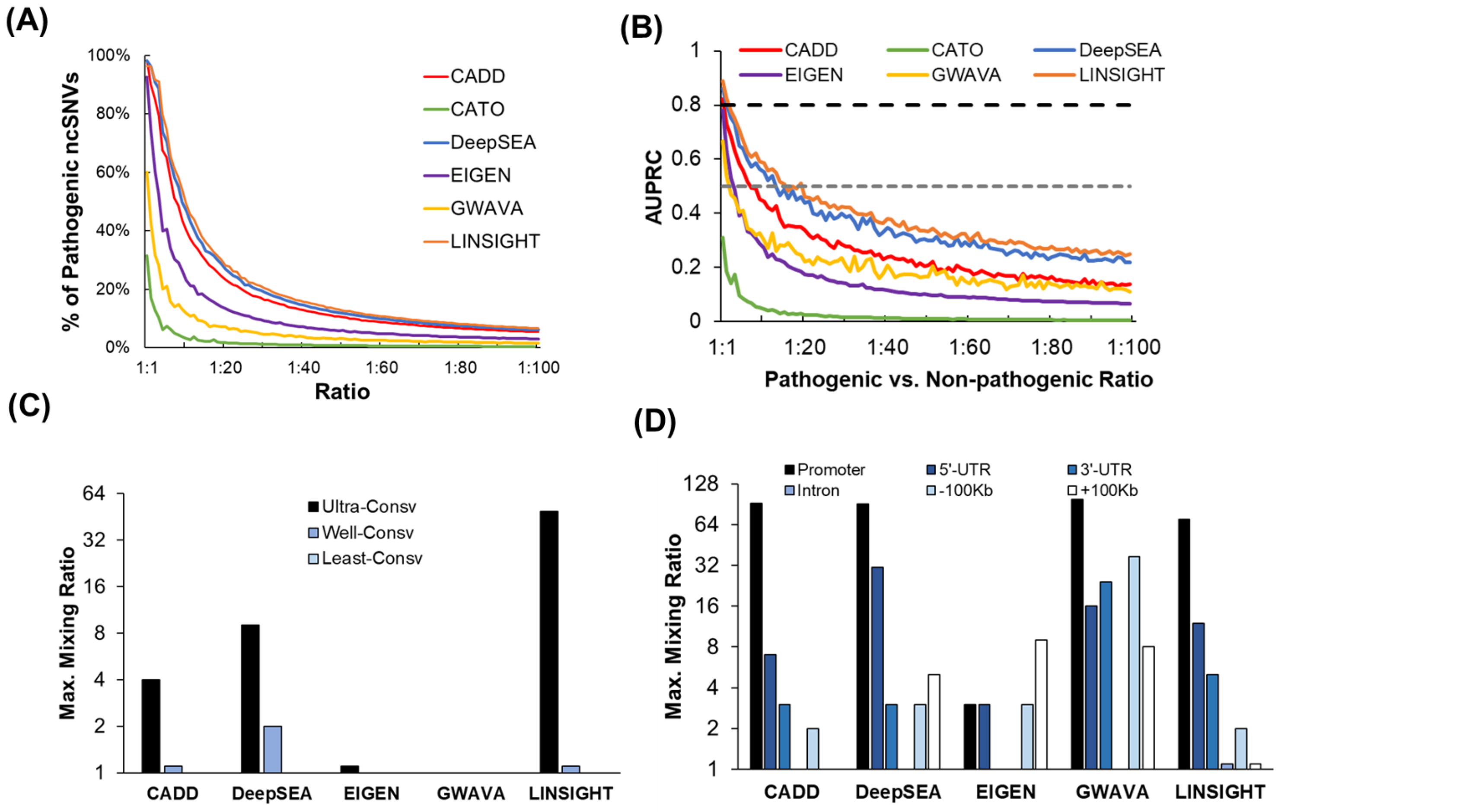

Supplementary Figure 9. Performance of tested methods on prioritizing ClinVar variants. Filtered ClinVar DAVs were used in the analysis. (**A**) Fraction of pathogenic ncSNVs in the top 10% percentile of impact scores declines exponentially as the mixing ratio increases. (**B**) AUPRC value decreases as the mixing ratio increases. (**C**) Maximum mixing ratio for each tool to achieve AUPRC > 0.8 when pathogenic ncSNVs disrupt ultra-, less-or least-conserved positions. (**D**) Maximum mixing ratio for each tool to achieve AUPRC > 0.8 when pathogenic ncSNVs is inside different types of genomic regions.

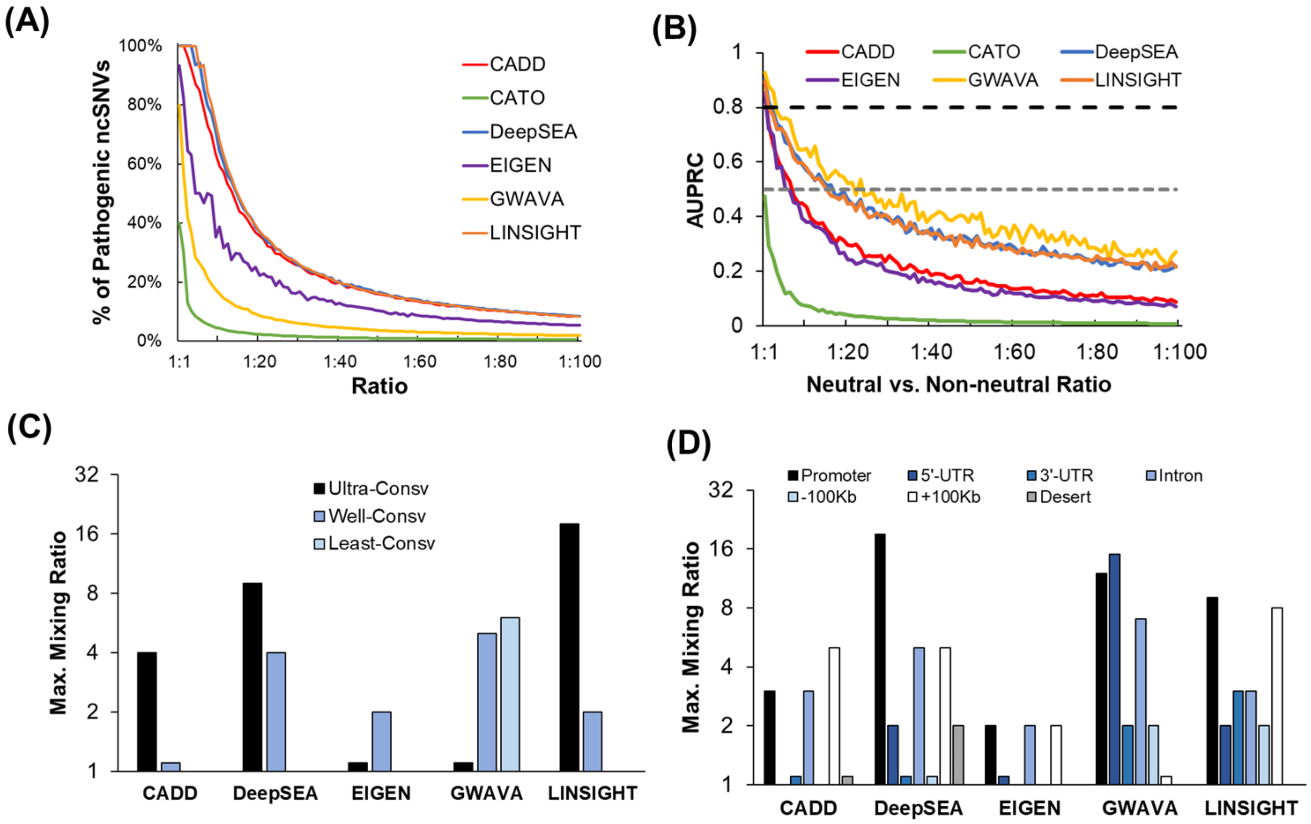

Supplementary Figure 10. Performance of tested methods on prioritizing unique HGMD GWAS hits. Only one DAV per gene was used in the analyses. (**A**) Fraction of pathogenic ncSNVs in the top 10% percentile of impact scores declines exponentially as the mixing ratio increases. (**B**) AUPRC value decreases as the mixing ratio increases. (**C**) Maximum mixing ratio for each tool to achieve AUPRC>0.8 when pathogenic ncSNVs disrupt ultra-, less-or least-conserved positions. (**D**) Maximum mixing ratio for each tool to achieve AUPRC>0.8 when pathogenic ncSNVs is inside different types of genomic regions.

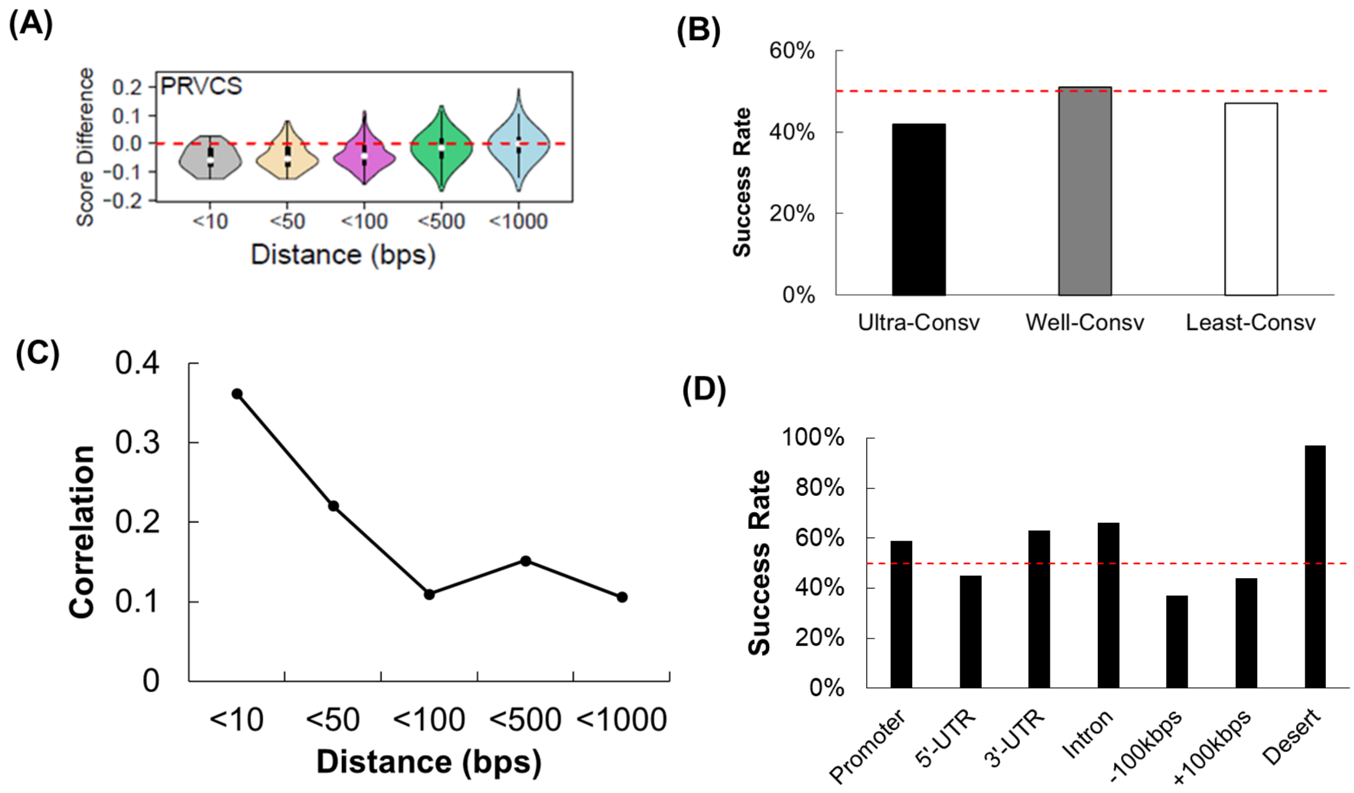

Supplementary Figure 11. Performance of PRVCS on diagnosing region-matched ncSNVs. (**A**) Violin plots show distributions of impact score difference between nearby pathogenic and non-pathogenic ncSNVs. Variants were grouped into bins based on distances measured by base pairs. (**B**) Correlation of impact scores for ncSNVs located within given genomic distances. (**C**) Success rate of ranking pathogenic ncSNVs to the top among non-pathogenic ncSNVs located within its 1,000 bps flanking region. Data are stratified by conservation of the position harboring pathogenic ncSNVs. The red dotted line represents the random expectation of success rate of 0.5. (**D**) Success rate stratified by the genomic context of pathogenic ncSNVs, including promoters, 5'-and 3'-untranslated regions (UTRs), introns, near-gene (+/-100 kbps) regions and gene-desert regions.

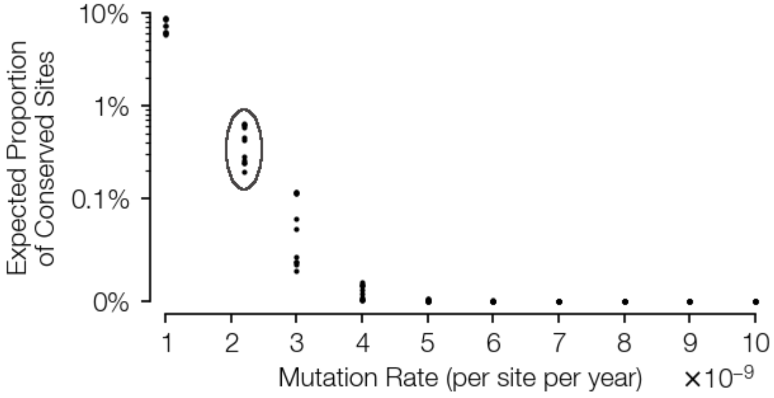

Supplementary Figure 12. Probability of observing completely conserved sites under strictly neutrality in a DNA sequence alignment of 58 placental mammals. For a given mutation rate along the x-axis, each point represents results from a given G+C content (θ) used for simulating sequences with the given mutation rate and the transition/transversion ratio (κ) of 3.6[66]. The maximum probability of observing a conserved site among the 58 placental mammals for sequences evolving with a mutation rate of 2.2×10-9 per site per year[65] and G+C content in the range of 10% - 90% (circled) is 0.6%.

Supplementary Table 1. The length of regions over which high LD with the candidate pathogenic variants is maintained does not vary meaningfully across the bins.

r2 range	# of SNP pairs	Genomic Distance (bps)		
		min	mean	max
>0.99	515	1	773	5,646
0.95 - 0.99	752	1	925	7,104
0.90 - 0.95	1,246	1	881	7,626
0.85 - 0.90	1,372	1	902	7,626
0.80 - 0.85	1,498	1	908	10,591

Supplementary Table 2. Cutoff score of each method, as determined by maximizing balanced accuracy (TPR + TNR) on DAV-vs-CPP dataset. TPR: true positive rate; TNR: true negative rate; BACC: balanced accuracy.

Method	Cutoff	TPR	TNR	BACC
CADD	6.40	0.76	0.84	0.80
LINSIGHT	0.08	0.82	0.87	0.84
EIGEN-PC	9.00	0.86	0.79	0.83
DeepSEA	0.05	0.80	0.88	0.84
GWAVA	0.66	0.93	0.92	0.93
CATO	0.27	0.01	0.99	0.50
PRVCS	0.03	0.87	0.80	0.84

Supplementary Table 3. HGMD variants passing three filters.

chrom	chrom_pos	reference	alternate	MAF (1kg)	MAF (GnomAD)	Nearest Gene	Region Type
1	8021918	C	G	0.00%	0.00%	ENSG00000116288	5'UTR
1	11083407	G	A	0.00%	0.00%	ENSG00000120948	3'UTR
1	11905783	T	G	0.00%	0.00%	ENSG00000175206	3'UTR
1	21835919	C	T	0.00%	0.00%	ENSG00000162551	5'UTR
1	25747286	G	A	0.00%	0.00%		5'UTR
1	26143315	T	C	0.00%	0.00%	ENSG00000117640	3'UTR
1	41223002	C	T	0.00%	0.00%	ENSG00000117013	Intron
1	55505179	C	A	0.00%	0.02%	ENSG00000169174	5'UTR
1	63788090	G	T	0.20%	0.06%	ENSG00000187140	100kb Upstream
1	92949057	T	G	0.00%	0.15%	ENSG00000162676	5'UTR
1	100661452	T	G	0.00%	0.00%	ENSG00000122477	3'UTR
1	101184876	A	G	0.20%	0.27%	ENSG00000162692	100kb Upstream
1	111220068	C	T	0.00%	0.14%	ENSG00000177272	100kb Upstream
1	113498813	C	T	0.00%	0.00%	ENSG00000155380	5'UTR
1	145507645	G	A	1.00%	1.67%	ENSG00000131779	5'UTR
1	145507764	G	C	0.10%	0.79%	ENSG00000131779	Intron
1	155211105	T	C	0.80%	0.86%	ENSG00000177628	promoter
1	155271257	T	C	0.00%	0.00%	ENSG00000143627	promoter
1	155271258	C	G	0.00%	0.00%	ENSG00000143627	promoter
1	155271268	C	G	0.00%	0.00%	ENSG00000143627	promoter
1	156028184	C	A	0.00%	0.00%	ENSG00000132698	3'UTR
1	159684664	G	A	0.00%	30.07%	ENSG00000132693	100kb Upstream
1	160001798	G	C	0.00%	0.00%	ENSG00000143315	promoter
1	161278298	A	T	0.00%	0.00%	ENSG00000158887	Intron
1	161632645	G	C	0.00%	0.00%	ENSG00000072694	100kb Upstream
1	161632911	T	A	0.00%	0.00%	ENSG00000072694	promoter
1	172628080	T	C	0.00%	0.00%	ENSG00000117560	100kb Upstream
1	173886567	G	C	0.00%	0.00%	ENSG00000117601	100kb Upstream
1	196620940	C	T	0.40%	0.69%	ENSG00000000971	100kb Upstream

1	204165662	G	A	0.00%	0.26%	ENSG00000170498	promoter
1	207494912	C	G	0.80%	0.89%	ENSG00000196352	5'UTR
1	209878285	G	A	0.00%	0.04%		5'UTR
1	209975331	G	T	0.00%	0.00%	ENSG00000117595	5'UTR
1	209975360	T	A	0.00%	0.00%	ENSG00000117595	5'UTR
1	209979366	C	T	0.00%	0.00%	ENSG00000117595	5'UTR
1	209979434	G	A	0.00%	0.00%	ENSG00000117595	5'UTR
1	216596609	C	A	0.00%	0.00%	ENSG00000042781	5'UTR
1	228337560	A	G	0.00%	0.00%	ENSG00000198835	5'UTR
2	25387651	G	T	0.00%	0.00%	ENSG00000115138	5'UTR
2	31639287	G	A	0.00%	0.01%	ENSG00000158125	100kb Upstream
2	38303242	T	G	0.00%	0.00%	ENSG00000138061	5'UTR
2	47630105	G	C	0.10%	0.01%	ENSG00000095002	100kb Upstream
2	47630149	G	A	0.00%	0.01%	ENSG00000095002	100kb Upstream
2	48034013	A	C	0.00%	0.00%	ENSG00000116062	3'UTR
2	49381592	T	C	0.00%	0.00%	ENSG00000170820	5'UTR
2	49381678	T	C	0.00%	0.00%	ENSG00000170820	promoter
2	49381693	T	A	0.00%	0.00%	ENSG00000170820	promoter
2	50574089	C	A	0.00%	0.00%	ENSG00000179915	Intron
2	71913728	T	A	0.50%	0.60%	ENSG00000135636	3'UTR
2	73114548	G	A	0.00%	0.00%	ENSG00000116096	5'UTR
2	96931136	G	A	0.00%	0.00%		5'UTR
2	122288484	G	A	0.00%	0.00%	ENSG00000074054	Intron
2	122288505	G	A	0.00%	0.04%	ENSG00000074054	Intron
2	122288509	G	A	0.00%	0.00%	ENSG00000074054	Intron
2	122288520	G	C	0.00%	0.00%	ENSG00000074054	Intron
2	122288565	G	A	0.00%	0.00%	ENSG00000074054	Intron
2	122288578	G	A	0.00%	0.00%	ENSG00000074054	Intron
2	128175982	A	G	0.00%	0.00%	ENSG00000115718	promoter
2	128175983	A	G	0.00%	0.00%	ENSG00000115718	promoter
2	128175987	T	A	0.00%	0.00%	ENSG00000115718	promoter
2	128175993	T	G	0.00%	0.00%	ENSG00000115718	promoter

2	128176000	T	C	0.00%	0.00%	ENSG00000115718	promoter
2	128176004	C	T	0.00%	0.00%	ENSG00000115718	promoter
2	128176035	G	A	0.00%	0.01%	ENSG00000115718	5'UTR
2	128176046	A	C	0.00%	0.00%	ENSG00000115718	5'UTR
2	128176057	G	A	0.00%	0.00%	ENSG00000115718	5'UTR
2	128186594	C	T	0.00%	0.00%	ENSG00000115718	3'UTR
2	131356866	T	C	0.00%	0.00%	ENSG00000136698	5'UTR
2	136608642	G	C	0.00%	0.00%	ENSG00000115850	Intron
2	136608650	A	C	0.10%	0.02%	ENSG00000115850	Intron
2	136608745	C	G	0.30%	0.00%	ENSG00000115850	Intron
2	157189109	A	C	0.00%	0.00%	ENSG00000153234	5'UTR
2	157189173	G	A	0.00%	0.00%	ENSG00000153234	5'UTR
2	203241528	G	A	0.30%	0.97%	ENSG00000204217	5'UTR
2	208394029	G	A	0.10%	0.19%	ENSG00000118260	100kb Upstream
2	208394570	A	G	0.20%	0.27%	ENSG00000118260	promoter
2	213404035	C	A	0.10%	0.00%	ENSG00000178568	100kb Upstream
2	213404068	T	A	0.10%	0.49%	ENSG00000178568	100kb Upstream
2	219028050	C	T	0.00%	0.02%	ENSG00000163464	3'UTR
2	219246417	G	C	0.20%	0.13%	ENSG00000018280	100kb Upstream
2	219246449	T	C	0.20%	1.51%	ENSG00000018280	100kb Upstream
3	9791917	G	C	0.20%	0.01%	ENSG00000114026	5'UTR
3	9791947	A	G	0.40%	0.07%	ENSG00000114026	5'UTR
3	9791952	G	T	0.70%	0.18%	ENSG00000114026	5'UTR
3	10191655	C	G	0.00%	0.00%	ENSG00000134086	3'UTR
3	11313448	G	A	0.20%	0.47%	ENSG00000197548	100kb Upstream
3	11313810	T	C	0.00%	0.00%	ENSG00000197548	100kb Upstream
3	11313912	G	A	0.00%	0.00%	ENSG00000197548	100kb Upstream
3	12421188	A	G	0.00%	0.01%	ENSG00000132170	Intron
3	14187284	C	T	0.00%	0.01%		3'UTR
3	15687153	G	A	0.00%	0.00%		3'UTR
3	37034931	C	G	0.00%	0.00%	ENSG00000076242	5'UTR
3	37034996	C	T	0.00%	0.01%	ENSG00000076242	5'UTR

3	37035011	C	A	0.00%	0.00%	ENSG00000076242	5'UTR
3	46412442	G	A	0.00%	0.03%	ENSG00000160791	5'UTR
3	47517343	G	A	0.10%	0.22%	ENSG00000114650	5'UTR
3	48632779	G	A	0.00%	0.00%	ENSG00000114270	100kb Upstream
3	49066877	A	C	0.00%	0.00%	ENSG00000178035	promoter
3	49209094	C	T	0.30%	0.52%	ENSG00000185909	5'UTR
3	50273449	C	G	0.00%	0.00%	ENSG00000114353	5'UTR
3	71834206	G	T	0.10%	0.08%	ENSG00000163421	5'UTR
3	93692596	G	C	0.00%	0.00%	ENSG00000184500	5'UTR
3	93692760	G	A	0.00%	0.00%	ENSG00000184500	5'UTR
3	115377645	G	T	0.00%	0.01%	ENSG00000172020	5'UTR
3	119500797	A	T	0.60%	0.20%	ENSG00000144852	5'UTR
3	119500973	C	A	0.20%	0.08%	ENSG00000144852	5'UTR
3	138663814	G	T	0.30%	0.30%		3'UTR
3	169482398	C	T	0.00%	0.00%	ENSG00000184378	100kb Downstream
3	169482440	G	C	0.00%	0.00%	ENSG00000184378	100kb Downstream
3	169482471	T	C	0.00%	0.00%	ENSG00000184378	100kb Downstream
3	169482523	C	A	0.00%	0.00%	ENSG00000184378	100kb Downstream
3	169482525	G	A	0.00%	0.00%	ENSG00000184378	100kb Downstream
3	169482526	C	T	0.00%	0.00%	ENSG00000184378	100kb Downstream
3	169482539	C	A	0.00%	0.00%	ENSG00000184378	100kb Downstream
3	169482543	C	T	0.00%	0.00%	ENSG00000184378	100kb Downstream
3	169482561	G	C	0.00%	0.00%	ENSG00000184378	100kb Downstream
3	169482604	G	A	0.00%	0.00%	ENSG00000184378	100kb Downstream
3	169482636	G	C	0.00%	0.00%	ENSG00000184378	100kb Downstream
3	169482644	G	C	0.00%	0.00%	ENSG00000184378	100kb Downstream
3	169482666	C	T	0.00%	0.00%	ENSG00000184378	100kb Downstream
3	169482668	G	A	0.00%	0.00%	ENSG00000184378	100kb Downstream
3	169482670	C	T	0.00%	0.00%	ENSG00000184378	100kb Downstream
3	169482672	T	G	0.00%	0.00%	ENSG00000184378	100kb Downstream
3	169482692	G	T	0.00%	0.00%	ENSG00000184378	100kb Downstream
3	169482705	C	T	0.00%	0.00%	ENSG00000184378	100kb Downstream

3	169482722	T	C	0.00%	0.00%	ENSG00000184378	100kb Downstream
3	169482731	T	G	0.00%	0.00%	ENSG00000184378	100kb Downstream
3	169482732	G	A	0.00%	0.00%	ENSG00000184378	100kb Downstream
3	169482741	C	A	0.00%	0.00%	ENSG00000184378	100kb Downstream
3	169482748	A	T	0.00%	0.00%	ENSG00000184378	100kb Downstream
3	169482750	C	T	0.00%	0.00%	ENSG00000184378	100kb Downstream
3	169482765	A	C	0.00%	0.00%	ENSG00000184378	100kb Downstream
3	169482776	G	C	0.00%	0.00%	ENSG00000184378	100kb Downstream
3	169482781	C	T	0.00%	0.00%	ENSG00000184378	100kb Downstream
3	169482800	T	C	0.00%	0.00%	ENSG00000184378	100kb Downstream
3	169482811	T	C	0.00%	0.00%	ENSG00000184378	100kb Downstream
3	169482812	G	A	0.00%	0.00%	ENSG00000184378	100kb Downstream
3	169482813	G	A	0.00%	0.00%	ENSG00000184378	100kb Downstream
3	169482846	C	G	0.00%	0.00%	ENSG00000184378	100kb Downstream
3	169482905	G	C	0.00%	0.00%	ENSG00000184378	100kb Downstream
3	169482946	G	C	0.00%	0.06%	ENSG00000184378	100kb Downstream
3	170745040	T	G	0.00%	0.00%	ENSG00000163581	100kb Upstream
3	184096786	T	C	0.00%	0.00%	ENSG00000090534	5'UTR
4	980870	C	G	0.00%	0.00%	ENSG00000127415	5'UTR
4	2963640	C	T	0.00%	0.00%		Intron
4	2963906	G	C	0.90%	1.19%		Intron
4	55161472	G	A	0.00%	0.00%	ENSG00000134853	3'UTR
4	74301816	G	A	0.00%	0.00%	ENSG00000081051	100kb Upstream
4	74301880	C	A	0.00%	0.00%	ENSG00000081051	100kb Upstream
4	100009922	G	A	0.00%	0.04%	ENSG00000197894	5'UTR
4	155513027	G	A	0.00%	0.00%	ENSG00000171560	100kb Upstream
4	187186994	G	A	0.00%	0.00%	ENSG00000088926	100kb Upstream
5	14871566	G	A	0.00%	0.00%	ENSG00000154122	5'UTR
5	36877265	C	T	0.00%	0.00%	ENSG00000164190	5'UTR
5	70220891	A	G	0.00%	0.00%	ENSG00000172062	5'UTR
5	79924682	G	A	0.00%	0.00%		3'UTR
5	95768972	C	A	0.50%	0.46%	ENSG00000175426	5'UTR

5	121413701	C	G	0.00%	0.00%	ENSG00000113083	5'UTR
5	131705345	C	A	0.30%	0.90%	ENSG00000197375	100kb Upstream
5	132009596	A	G	0.00%	0.00%	ENSG00000113520	100kb Upstream
5	142782401	C	T	0.00%	16.19%	ENSG00000113580	5'UTR
5	142782980	G	T	0.10%	0.11%	ENSG00000113580	5'UTR
5	142783267	A	G	0.20%	0.38%	ENSG00000113580	promoter
5	147211192	G	A	0.00%	0.02%	ENSG00000164266	5'UTR
5	147211354	C	T	0.10%	0.02%	ENSG00000164266	5'UTR
5	148203700	A	G	0.00%	0.00%	ENSG00000169252	100kb Upstream
5	150019897	C	T	0.00%	0.00%	ENSG00000171992	Intron
5	156607792	C	T	0.40%	0.06%		100kb Upstream
5	161274805	A	C	0.30%	0.15%	ENSG00000022355	5'UTR
5	172662541	G	A	0.00%	0.00%	ENSG00000183072	100kb Upstream
5	176836589	G	A	0.10%	0.31%	ENSG00000131187	promoter
6	7586119	T	A	0.30%	0.52%	ENSG00000168566	3'UTR
6	10398115	T	G	0.00%	0.00%	ENSG00000137203	3'UTR
6	26087648	G	A	0.00%	0.00%	ENSG00000010704	5'UTR
6	29910229	T	C	0.00%	0.00%	ENSG00000206503	promoter
6	31795274	C	T	0.30%	0.64%	ENSG00000204388	100kb Upstream
6	32097632	T	C	0.00%	0.00%	ENSG00000204315	5'UTR
6	32908751	G	A	0.50%	0.80%	ENSG00000242574	5'UTR
6	32908815	G	A	0.20%	0.37%	ENSG00000242574	5'UTR
6	52668733	C	T	0.10%	0.04%	ENSG00000243955	5'UTR
6	118869381	A	G	0.00%	0.00%	ENSG00000198523	promoter
6	118869416	C	G	0.00%	0.01%	ENSG00000198523	promoter
6	132272534	G	C	0.00%	0.00%	ENSG00000118523	promoter
6	137143758	C	T	0.00%	0.00%	ENSG00000112357	5'UTR
6	152420196	C	T	0.00%	0.16%		3'UTR
6	154360126	G	A	0.00%	0.09%	ENSG00000112038	5'UTR
6	163148720	C	A	0.00%	0.00%		5'UTR
7	6414150	C	G	0.00%	0.27%	ENSG00000136238	5'UTR
7	6414152	A	T	0.00%	0.00%	ENSG00000136238	5'UTR

7	22766541	A	C	0.00%	0.00%	ENSG00000136244	100kb Upstream
7	31003517	T	C	0.00%	0.00%		100kb Upstream
7	31003519	T	C	0.00%	0.01%		100kb Upstream
7	31003559	A	C	0.00%	0.00%		100kb Upstream
7	35293779	C	T	0.00%	0.00%	ENSG00000164532	100kb Upstream
7	44229108	C	G	0.00%	0.00%		100kb Upstream
7	44836229	C	G	0.00%	0.00%	ENSG00000196262	promoter
7	73137971	T	A	0.00%	0.00%	ENSG00000106089	100kb Upstream
7	75931812	T	C	0.00%	0.00%	ENSG00000106211	100kb Upstream
7	87238033	G	A	0.10%	0.03%		5'UTR
7	96641428	A	G	0.00%	0.00%	ENSG00000006377	100kb Downstream
7	99277537	A	G	0.00%	0.02%	ENSG00000106258	5'UTR
7	99277989	A	C	0.00%	0.00%	ENSG00000106258	100kb Upstream
7	99354787	C	T	0.00%	0.00%	ENSG00000160870	3'UTR
7	107301200	T	C	0.00%	0.21%	ENSG00000091137	5'UTR
7	107301243	A	G	0.00%	0.04%	ENSG00000091137	5'UTR
7	107355959	C	A	0.30%	0.11%	ENSG00000105879	3'UTR
7	117119653	C	T	0.00%	0.00%	ENSG00000001626	100kb Upstream
7	117119687	A	G	0.10%	0.37%	ENSG00000001626	100kb Upstream
7	117119922	G	T	0.40%	0.38%	ENSG00000001626	100kb Upstream
7	117119983	G	A	0.00%	0.01%	ENSG00000001626	promoter
7	117120063	C	G	0.00%	0.00%	ENSG00000001626	5'UTR
7	117120114	C	T	0.00%	0.00%	ENSG00000001626	5'UTR
7	129414552	A	G	0.00%	0.01%	ENSG00000106459	100kb Downstream
7	129414595	G	T	0.00%	0.00%	ENSG00000106459	100kb Downstream
7	129414596	C	T	0.00%	0.00%	ENSG00000106459	100kb Downstream
7	134144017	G	T	0.00%	0.00%	ENSG00000085662	promoter
7	142959574	G	C	0.00%	3.39%	ENSG00000197448	100kb Upstream
7	156061505	C	T	0.00%	0.00%	ENSG00000204876	desert
7	156583830	T	C	0.00%	0.00%	ENSG00000105983	Intron
7	156583948	G	C	0.00%	0.00%	ENSG00000105983	Intron
7	156583950	G	A	0.00%	0.00%	ENSG00000105983	Intron

7	156584106	A	C	0.00%	0.00%	ENSG00000105983	Intron
7	156584165	C	T	0.00%	0.00%	ENSG00000105983	Intron
7	156584240	A	G	0.00%	0.00%	ENSG00000105983	Intron
7	156584264	T	A	0.00%	0.00%	ENSG00000105983	Intron
7	156584274	A	G	0.00%	0.00%	ENSG00000105983	Intron
7	156584282	G	T	0.00%	0.00%	ENSG00000105983	Intron
7	156584464	G	C	0.00%	0.00%	ENSG00000105983	Intron
7	156584862	G	A	0.00%	0.00%	ENSG00000105983	Intron
8	11331746	G	A	0.00%	0.01%	ENSG00000154319	100kb Upstream
8	11421954	T	G	0.40%	0.32%	ENSG00000136573	3'UTR
8	11422121	G	T	0.00%	0.00%	ENSG00000136573	100kb Downstream
8	11560786	T	C	0.10%	0.03%	ENSG00000136574	5'UTR
8	11560863	C	A	0.00%	0.03%	ENSG00000136574	5'UTR
8	11561281	C	T	0.00%	0.00%	ENSG00000136574	5'UTR
8	11561368	G	T	0.00%	0.00%	ENSG00000136574	5'UTR
8	17358052	A	G	0.00%	0.02%	ENSG00000003989	5'UTR
8	19796710	G	C	0.00%	0.07%	ENSG00000175445	5'UTR
8	19796724	T	C	0.00%	0.00%	ENSG00000175445	5'UTR
8	22016931	G	T	0.60%	0.96%		100kb Upstream
8	22017669	G	A	0.60%	0.98%		100kb Upstream
8	22018139	A	G	0.00%	0.00%		100kb Upstream
8	38008507	G	A	0.80%	1.37%	ENSG00000147465	5'UTR
8	53627493	G	A	0.00%	0.01%	ENSG00000023287	100kb Upstream
8	53627572	A	G	0.00%	0.01%	ENSG00000023287	100kb Upstream
8	82395534	A	G	0.60%	2.10%	ENSG00000170323	100kb Upstream
8	104343756	C	T	0.00%	0.00%	ENSG00000164930	3'UTR
8	108262668	T	A	0.00%	34.20%	ENSG00000154188	3'UTR
8	119124442	C	G	0.00%	3.96%	ENSG00000182197	100kb Upstream
9	21974846	G	A	0.00%	0.00%	ENSG00000224854	promoter
9	21974850	G	A	0.90%	1.11%	ENSG00000224854	promoter
9	21974859	C	G	0.00%	0.03%	ENSG00000224854	promoter
9	21974874	G	T	0.00%	0.00%	ENSG00000224854	promoter

9	21974881	C	A	0.00%	0.00%	ENSG00000224854	5'UTR
9	21974892	C	G	0.00%	0.00%	ENSG00000224854	5'UTR
9	22010003	T	A	0.00%	30.97%	ENSG00000147883	100kb Upstream
9	34635577	C	A	0.00%	0.00%	ENSG00000147955	3'UTR
9	34638233	A	T	0.20%	0.07%	ENSG00000147955	100kb Upstream
9	35657744	A	G	0.00%	0.01%	ENSG00000159884	100kb Upstream
9	35657750	G	T	0.00%	0.00%	ENSG00000159884	100kb Upstream
9	35657752	C	A	0.00%	0.01%	ENSG00000159884	100kb Upstream
9	35657753	G	A	0.00%	0.00%	ENSG00000159884	100kb Upstream
9	35657760	G	C	0.00%	0.00%	ENSG00000159884	100kb Upstream
9	35657762	A	C	0.00%	0.00%	ENSG00000159884	100kb Upstream
9	35657766	G	A	0.00%	0.00%	ENSG00000159884	100kb Upstream
9	35657770	C	T	0.00%	0.00%	ENSG00000159884	100kb Upstream
9	35657771	G	A	0.00%	0.00%	ENSG00000159884	100kb Upstream
9	35657772	T	C	0.00%	0.01%	ENSG00000159884	100kb Upstream
9	35657774	T	G	0.00%	0.00%	ENSG00000159884	100kb Upstream
9	35657776	G	A	0.00%	0.01%	ENSG00000159884	100kb Upstream
9	35657778	T	C	0.00%	0.00%	ENSG00000159884	100kb Upstream
9	35657784	G	A	0.00%	0.00%	ENSG00000159884	100kb Upstream
9	35657794	A	G	0.00%	0.00%	ENSG00000159884	100kb Upstream
9	35657796	T	C	0.00%	0.00%	ENSG00000159884	100kb Upstream
9	35657797	G	A	0.00%	0.00%	ENSG00000159884	100kb Upstream
9	35657800	T	A	0.00%	0.01%	ENSG00000159884	100kb Upstream
9	35657801	G	C	0.00%	0.00%	ENSG00000159884	100kb Upstream
9	35657803	G	C	0.00%	0.00%	ENSG00000159884	100kb Upstream
9	35657819	G	A	0.00%	0.00%	ENSG00000159884	100kb Upstream
9	35657821	C	T	0.00%	0.00%	ENSG00000159884	100kb Upstream
9	35657832	C	G	0.00%	0.00%	ENSG00000159884	100kb Upstream
9	35657834	C	T	0.00%	0.00%	ENSG00000159884	100kb Upstream
9	35657846	C	T	0.00%	0.00%	ENSG00000159884	100kb Upstream
9	35657860	C	A	0.00%	0.00%	ENSG00000159884	100kb Upstream
9	35657862	T	C	0.00%	0.00%	ENSG00000159884	100kb Upstream

9	35657868	C	T	0.00%	0.00%	ENSG00000159884	100kb Upstream
9	35657887	C	T	0.30%	0.00%	ENSG00000159884	100kb Upstream
9	35657888	G	A	0.00%	0.00%	ENSG00000159884	100kb Upstream
9	35657890	G	A	0.00%	0.00%	ENSG00000159884	100kb Upstream
9	35657896	T	C	0.00%	0.00%	ENSG00000159884	100kb Upstream
9	35657898	T	C	0.00%	0.01%	ENSG00000159884	100kb Upstream
9	35657913	G	A	0.00%	0.00%	ENSG00000159884	100kb Upstream
9	35657915	G	A	0.00%	0.01%	ENSG00000159884	100kb Upstream
9	35657917	C	T	0.00%	0.00%	ENSG00000159884	100kb Upstream
9	35657921	C	G	0.00%	0.00%	ENSG00000159884	100kb Upstream
9	35657923	C	T	0.00%	0.00%	ENSG00000159884	100kb Upstream
9	35657925	G	C	0.00%	0.00%	ENSG00000159884	100kb Upstream
9	35657934	C	T	0.00%	0.00%	ENSG00000159884	100kb Upstream
9	35657935	C	T	0.00%	0.00%	ENSG00000159884	100kb Upstream
9	35657937	G	A	0.00%	0.00%	ENSG00000159884	100kb Upstream
9	35657944	T	C	0.00%	0.15%	ENSG00000159884	100kb Upstream
9	35657950	A	G	0.00%	0.00%	ENSG00000159884	100kb Upstream
9	35657951	G	A	0.00%	0.00%	ENSG00000159884	100kb Upstream
9	35657953	C	T	0.00%	0.00%	ENSG00000159884	100kb Upstream
9	35657974	C	T	0.00%	0.00%	ENSG00000159884	100kb Upstream
9	35657979	G	A	0.00%	0.01%	ENSG00000159884	100kb Upstream
9	35657987	C	T	0.00%	0.00%	ENSG00000159884	100kb Upstream
9	35657996	C	G	0.00%	0.00%	ENSG00000159884	100kb Upstream
9	35658000	C	T	0.00%	0.00%	ENSG00000159884	100kb Upstream
9	35658003	T	C	0.00%	0.00%	ENSG00000159884	100kb Upstream
9	35658005	A	G	0.00%	0.00%	ENSG00000159884	100kb Upstream
9	35658010	G	A	0.00%	0.02%	ENSG00000159884	100kb Upstream
9	90107461	A	G	0.00%	0.04%	ENSG00000196730	100kb Upstream
9	104198193	C	T	0.50%	0.60%	ENSG00000136872	100kb Upstream
9	107690401	G	C	0.90%	2.46%	ENSG00000165029	5'UTR
9	120477932	G	C	0.60%	0.20%	ENSG00000136869	3'UTR
9	130548228	C	G	0.00%	0.00%	ENSG00000136807	100kb Upstream

9	130616760	G	A	0.00%	0.00%	ENSG00000106991	5'UTR
9	133327611	C	T	0.00%	0.02%	ENSG00000130707	5'UTR
10	6104741	G	A	0.20%	0.25%	ENSG00000134460	100kb Upstream
10	27389367	T	G	0.00%	0.02%	ENSG00000107890	5'UTR
10	27389373	G	T	0.00%	0.00%	ENSG00000107890	5'UTR
10	27389375	T	G	0.00%	0.00%	ENSG00000107890	5'UTR
10	27389380	A	C	0.00%	0.00%	ENSG00000107890	5'UTR
10	27389381	T	A	0.00%	0.00%	ENSG00000107890	5'UTR
10	27389382	C	T	0.00%	0.00%	ENSG00000107890	5'UTR
10	27389388	C	T	0.00%	0.00%	ENSG00000107890	5'UTR
10	43572511	G	A	0.00%	0.00%	ENSG00000165731	promoter
10	43572515	C	A	0.00%	0.00%	ENSG00000165731	promoter
10	43572669	G	C	0.00%	0.00%	ENSG00000165731	5'UTR
10	43572679	C	G	0.00%	0.00%	ENSG00000165731	5'UTR
10	71075517	A	G	0.10%	0.29%		Intron
10	89622987	A	G	0.00%	0.00%	ENSG00000171862	5'UTR
10	89623048	C	T	0.00%	0.00%	ENSG00000171862	5'UTR
10	89623055	C	T	0.00%	0.00%	ENSG00000171862	5'UTR
10	89623083	C	T	0.20%	0.04%	ENSG00000171862	5'UTR
10	89623115	A	G	0.00%	0.00%	ENSG00000171862	5'UTR
10	89623141	C	T	0.60%	0.25%	ENSG00000171862	5'UTR
10	89623225	T	C	0.00%	0.00%	ENSG00000171862	promoter
10	89623295	G	A	0.00%	0.00%	ENSG00000171862	5'UTR
10	89623305	G	T	0.00%	0.00%	ENSG00000171862	5'UTR
10	89623330	T	C	0.00%	0.00%	ENSG00000171862	5'UTR
10	89623364	G	T	0.00%	0.00%	ENSG00000171862	5'UTR
10	89623372	C	G	0.00%	0.00%	ENSG00000171862	5'UTR
10	89623391	C	T	0.00%	0.00%	ENSG00000171862	5'UTR
10	89623427	G	C	0.00%	0.00%	ENSG00000171862	5'UTR
10	89725293	T	A	0.00%	0.00%	ENSG00000171862	3'UTR
10	96520964	T	G	0.00%	0.00%	ENSG00000165841	100kb Upstream
10	96521020	T	C	0.00%	0.00%	ENSG00000165841	100kb Upstream

10	96521683	A	C	0.00%	0.00%	ENSG00000165841	100kb Upstream
10	96698277	A	G	0.00%	0.00%	ENSG00000138109	100kb Upstream
10	127505270	A	G	0.00%	0.00%		5'UTR
10	127505276	C	T	0.00%	0.00%		5'UTR
10	127505286	G	T	0.00%	0.00%		5'UTR
10	127505290	G	T	0.00%	0.00%		5'UTR
11	299503	G	A	0.00%	0.00%	ENSG00000206013	5'UTR
11	2020123	C	A	0.00%	0.00%	ENSG00000130595	100kb Downstream
11	2023018	A	G	0.00%	0.00%	ENSG00000130595	100kb Downstream
11	2023047	A	C	0.00%	0.00%	ENSG00000130595	100kb Downstream
11	2181022	T	C	0.00%	0.00%		3'UTR
11	2182418	T	G	0.00%	0.00%		5'UTR
11	2182531	G	C	0.00%	0.01%		100kb Upstream
11	2182532	G	C	0.00%	0.03%		100kb Upstream
11	2193084	A	T	0.00%	0.00%	ENSG00000180176	promoter
11	2193085	C	T	0.00%	0.00%	ENSG00000180176	promoter
11	2193086	G	A	0.00%	0.00%	ENSG00000180176	100kb Upstream
11	2905208	C	A	0.00%	0.00%	ENSG00000129757	3'UTR
11	2906802	C	T	0.40%	0.48%	ENSG00000129757	5'UTR
11	5246695	G	T	0.00%	0.00%	ENSG00000244734	3'UTR
11	5246698	A	G	0.10%	0.00%	ENSG00000244734	3'UTR
11	5246714	T	C	0.00%	0.00%	ENSG00000244734	3'UTR
11	5246715	T	A	0.00%	0.00%	ENSG00000244734	3'UTR
11	5246716	T	C	0.00%	0.00%	ENSG00000244734	3'UTR
11	5246717	A	C	0.00%	0.00%	ENSG00000244734	3'UTR
11	5246719	T	G	0.00%	0.00%	ENSG00000244734	3'UTR
11	5246731	A	G	0.00%	0.27%	ENSG00000244734	3'UTR
11	5246753	T	C	0.00%	0.00%	ENSG00000244734	3'UTR
11	5246780	G	C	0.00%	0.00%	ENSG00000244734	3'UTR
11	5246795	T	G	0.00%	0.00%	ENSG00000244734	3'UTR
11	5246821	G	C	0.00%	0.00%	ENSG00000244734	3'UTR
11	5248256	C	G	0.00%	0.00%	ENSG00000244734	5'UTR

11	5248262	G	A	0.00%	0.00%	ENSG00000244734	5'UTR
11	5248268	G	C	0.00%	0.00%	ENSG00000244734	5'UTR
11	5248279	C	T	0.00%	0.00%	ENSG00000244734	5'UTR
11	5248281	G	A	0.00%	0.00%	ENSG00000244734	5'UTR
11	5248293	G	A	0.00%	0.00%	ENSG00000244734	promoter
11	5248300	T	C	0.00%	0.00%	ENSG00000244734	promoter
11	5248325	C	G	0.00%	0.00%	ENSG00000244734	promoter
11	5248326	T	G	0.00%	0.00%	ENSG00000244734	promoter
11	5248327	T	C	0.00%	0.00%	ENSG00000244734	promoter
11	5248328	T	G	0.00%	0.00%	ENSG00000244734	promoter
11	5248329	T	C	0.00%	0.09%	ENSG00000244734	promoter
11	5248330	A	T	0.00%	0.00%	ENSG00000244734	promoter
11	5248331	T	G	0.00%	0.00%	ENSG00000244734	promoter
11	5248332	G	A	0.00%	0.00%	ENSG00000244734	promoter
11	5248341	T	G	0.00%	0.00%	ENSG00000244734	promoter
11	5248342	G	C	0.00%	0.00%	ENSG00000244734	promoter
11	5248350	C	T	0.10%	0.00%	ENSG00000244734	promoter
11	5248356	C	G	0.00%	0.04%	ENSG00000244734	100kb Upstream
11	5248371	G	A	0.00%	0.00%	ENSG00000244734	100kb Upstream
11	5248373	T	A	0.00%	0.00%	ENSG00000244734	100kb Upstream
11	5248376	G	T	0.00%	0.00%	ENSG00000244734	100kb Upstream
11	5248383	C	T	0.20%	0.27%	ENSG00000244734	100kb Upstream
11	5248386	G	A	0.00%	0.00%	ENSG00000244734	100kb Upstream
11	5248387	G	C	0.00%	0.00%	ENSG00000244734	100kb Upstream
11	5248388	G	A	0.00%	0.03%	ENSG00000244734	100kb Upstream
11	5248390	G	A	0.00%	0.01%	ENSG00000244734	100kb Upstream
11	5248392	G	A	0.00%	0.00%	ENSG00000244734	100kb Upstream
11	5248393	G	C	0.00%	0.00%	ENSG00000244734	100kb Upstream
11	5248401	G	A	0.00%	0.00%	ENSG00000244734	100kb Upstream
11	5248402	G	T	0.00%	0.00%	ENSG00000244734	100kb Upstream
11	5248490	C	T	0.00%	0.00%	ENSG00000244734	100kb Upstream
11	5253994	C	T	0.00%	0.00%	ENSG00000223609	100kb Downstream

11	5254084	T	A	0.00%	0.00%	ENSG00000223609	3'UTR
11	5255742	A	G	0.00%	0.00%	ENSG00000223609	5'UTR
11	5255743	T	C	0.00%	0.00%	ENSG00000223609	5'UTR
11	5255748	G	T	0.00%	0.00%	ENSG00000223609	5'UTR
11	5255767	A	G	0.00%	0.01%	ENSG00000223609	5'UTR
11	5255777	T	C	0.00%	0.01%	ENSG00000223609	5'UTR
11	5255780	G	A	0.40%	0.00%	ENSG00000223609	5'UTR
11	5255788	T	A	0.00%	0.00%	ENSG00000223609	5'UTR
11	5255789	A	G	0.10%	0.01%	ENSG00000223609	5'UTR
11	5255792	C	T	0.00%	0.00%	ENSG00000223609	5'UTR
11	5268680	G	A	0.30%	0.08%	ENSG00000213934	100kb Downstream
11	5271200	G	A	0.00%	0.00%	ENSG00000213934	100kb Upstream
11	5271203	C	T	0.00%	0.00%	ENSG00000213934	100kb Upstream
11	5271261	A	G	0.00%	0.00%	ENSG00000213934	100kb Upstream
11	5271281	G	C	0.00%	0.00%	ENSG00000213934	100kb Upstream
11	5271282	G	A	0.00%	0.01%	ENSG00000213934	100kb Upstream
11	5271284	A	G	0.00%	0.00%	ENSG00000213934	100kb Upstream
11	5271287	G	A	0.00%	0.00%	ENSG00000213934	100kb Upstream
11	5271288	G	A	0.00%	0.00%	ENSG00000213934	100kb Upstream
11	5276119	C	A	0.00%	0.00%		100kb Upstream
11	5276124	G	A	0.00%	0.00%		100kb Upstream
11	5276168	G	A	0.00%	20.75%		100kb Upstream
11	5276171	C	T	0.00%	0.02%		100kb Upstream
11	5276185	A	G	0.00%	0.00%		100kb Upstream
11	5276206	G	A	0.00%	0.00%		100kb Upstream
11	5276212	G	C	0.00%	0.00%		100kb Upstream
11	5276577	A	C	0.00%	0.02%		100kb Upstream
11	17409691	G	A	0.00%	0.01%	ENSG00000187486	5'UTR
11	17409771	C	A	0.00%	0.00%	ENSG00000187486	5'UTR
11	17498512	G	C	0.00%	0.00%	ENSG00000006071	100kb Upstream
11	27743555	G	T	0.70%	1.91%	ENSG00000176697	5'UTR
11	46761053	C	T	0.10%	0.08%	ENSG00000180210	3'UTR

11	46761054	G	A	0.40%	0.84%	ENSG00000180210	3'UTR
11	46761063	T	A	0.00%	0.00%	ENSG00000180210	100kb Downstream
11	46761065	C	T	0.00%	0.00%	ENSG00000180210	100kb Downstream
11	47470714	G	C	0.00%	0.00%	ENSG00000165917	5'UTR
11	47470725	T	C	0.00%	0.00%	ENSG00000165917	promoter
11	57365054	C	T	0.00%	0.00%	ENSG00000149131	5'UTR
11	57365056	A	G	0.00%	0.00%	ENSG00000149131	5'UTR
11	57365117	C	G	0.00%	0.39%	ENSG00000149131	5'UTR
11	61735060	T	A	0.00%	0.00%	ENSG00000167996	5'UTR
11	62009280	G	A	0.00%	0.00%	ENSG00000124935	100kb Upstream
11	64577602	G	T	0.00%	0.12%	ENSG00000133895	5'UTR
11	76839533	A	G	0.00%	0.00%	ENSG00000137474	5'UTR
11	88070894	G	T	0.00%	0.00%	ENSG00000109861	5'UTR
11	93466908	A	G	0.00%	0.69%		100kb Downstream
11	104905938	G	A	0.00%	0.00%	ENSG00000137752	100kb Upstream
11	108093769	A	G	0.00%	0.00%		5'UTR
11	108094507	G	A	0.00%	0.00%		5'UTR
11	116708298	C	T	0.00%	0.00%	ENSG00000118137	promoter
11	116708364	T	G	0.00%	0.00%	ENSG00000118137	promoter
11	118955412	C	T	0.00%	0.01%	ENSG00000256269	100kb Upstream
11	118955473	G	A	0.00%	0.00%	ENSG00000256269	100kb Upstream
11	118955621	T	A	0.00%	0.00%	ENSG00000256269	5'UTR
11	118955715	A	C	0.10%	0.33%	ENSG00000256269	5'UTR
11	124609360	A	G	0.00%	0.00%	ENSG00000154146	100kb Upstream
11	124609402	C	G	0.00%	0.00%	ENSG00000154146	100kb Upstream
11	124609636	G	A	0.10%	0.00%	ENSG00000154146	100kb Upstream
11	124609906	C	G	0.00%	0.00%	ENSG00000154146	5'UTR
11	124609939	G	A	0.00%	0.00%	ENSG00000154146	5'UTR
12	6234257	G	A	0.20%	0.08%	ENSG00000110799	100kb Upstream
12	6235093	G	A	0.00%	0.00%	ENSG00000110799	100kb Upstream
12	12870766	G	C	0.00%	0.00%	ENSG00000111276	5'UTR
12	53715653	G	A	0.00%	0.00%	ENSG00000094914	100kb Upstream

12	68553702	C	A	0.60%	0.73%	ENSG00000111537	100kb Upstream
12	103232808	T	C	0.30%	0.01%		3'UTR
12	110718410	G	T	0.00%	0.00%	ENSG00000174437	100kb Upstream
12	110718566	G	T	0.00%	0.00%	ENSG00000174437	100kb Upstream
12	110719584	C	G	0.40%	0.67%	ENSG00000174437	5'UTR
12	114704514	G	T	0.10%	0.05%	ENSG00000089225	100kb Downstream
12	121416033	G	C	0.00%	0.00%	ENSG00000135100	100kb Upstream
12	121416109	G	A	0.00%	0.00%	ENSG00000135100	100kb Upstream
12	121416288	A	C	0.00%	0.00%	ENSG00000135100	100kb Upstream
12	121416353	T	C	0.00%	0.00%	ENSG00000135100	promoter
12	121416384	C	T	0.00%	0.00%	ENSG00000135100	5'UTR
12	121416443	T	G	0.00%	0.00%	ENSG00000135100	5'UTR
12	121416447	G	C	0.10%	0.08%	ENSG00000135100	5'UTR
12	121416452	G	A	0.00%	0.01%	ENSG00000135100	5'UTR
12	121416474	T	G	0.00%	0.00%	ENSG00000135100	5'UTR
12	121416509	C	G	0.00%	0.00%	ENSG00000135100	5'UTR
13	20767157	G	A	0.00%	0.00%	ENSG00000165474	promoter
13	29069941	G	A	0.80%	1.99%	ENSG00000102755	100kb Upstream
13	46679457	C	T	0.70%	1.51%	ENSG00000080618	100kb Upstream
13	48877836	G	A	0.00%	0.00%	ENSG00000139687	promoter
13	48877850	G	A	0.00%	0.00%	ENSG00000139687	promoter
13	48877855	T	A	0.00%	0.00%	ENSG00000139687	promoter
13	48877859	G	T	0.00%	0.00%	ENSG00000139687	promoter
13	48877898	G	C	0.00%	0.00%	ENSG00000139687	5'UTR
13	48877899	G	T	0.00%	0.00%	ENSG00000139687	5'UTR
13	48878044	C	G	0.00%	0.00%	ENSG00000139687	5'UTR
13	50623101	G	A	0.00%	0.00%	ENSG00000176124	100kb Downstream
13	52585550	T	G	0.00%	0.00%		5'UTR
13	52585595	G	T	0.00%	0.00%		5'UTR
13	52585605	T	G	0.00%	0.00%		5'UTR
13	52585682	T	A	0.00%	0.01%		100kb Upstream
13	60738071	C	T	0.00%	0.00%	ENSG00000139734	5'UTR

13	84452862	C	T	0.10%	0.00%	ENSG00000178235	3'UTR
13	92002950	C	T	0.00%	0.00%	ENSG00000179399	100kb Upstream
13	100634294	C	T	0.00%	0.00%	ENSG00000043355	5'UTR
13	113760059	C	T	0.00%	0.00%	ENSG00000057593	promoter
13	113760061	C	G	0.00%	0.00%	ENSG00000057593	promoter
13	113760090	G	C	0.00%	0.00%	ENSG00000057593	promoter
13	113760093	C	T	0.00%	0.00%	ENSG00000057593	promoter
13	113760094	T	G	0.00%	0.00%	ENSG00000057593	promoter
13	113760095	T	C	0.00%	0.00%	ENSG00000057593	promoter
13	113760096	T	G	0.00%	0.00%	ENSG00000057593	promoter
13	113760100	C	T	0.00%	0.00%	ENSG00000057593	promoter
13	113760111	T	C	0.10%	0.00%	ENSG00000057593	promoter
13	113760116	A	G	0.00%	0.00%	ENSG00000057593	5'UTR
13	113760123	A	C	0.00%	0.04%	ENSG00000057593	5'UTR
13	113760125	A	C	0.00%	0.00%	ENSG00000057593	5'UTR
13	113760154	C	T	0.00%	0.00%	ENSG00000057593	5'UTR
14	21162168	C	T	0.00%	0.00%	ENSG00000214274	5'UTR
14	21162172	G	A	0.00%	0.01%	ENSG00000214274	5'UTR
14	37130035	G	A	0.00%	0.00%	ENSG00000198807	5'UTR
14	55369402	G	A	0.00%	0.00%	ENSG00000131979	5'UTR
14	55369419	G	A	0.00%	0.00%	ENSG00000131979	5'UTR
14	64761120	A	C	0.90%	0.64%	ENSG00000140009	promoter
14	73600360	A	G	0.00%	0.08%	ENSG00000080815	100kb Upstream
14	73602898	C	G	0.30%	0.26%	ENSG00000080815	100kb Upstream
14	73603503	G	A	0.00%	0.03%	ENSG00000080815	5'UTR
14	76425034	G	A	0.00%	0.00%	ENSG00000119699	3'UTR
14	76447265	C	T	0.00%	0.00%	ENSG00000119699	5'UTR
14	82000441	A	G	0.20%	0.13%	ENSG00000071537	100kb Upstream
14	82000453	A	G	0.20%	0.13%	ENSG00000071537	100kb Upstream
15	34631467	T	C	0.60%	0.88%	ENSG00000182117	100kb Upstream
15	39880673	G	A	0.00%	0.25%	ENSG00000137801	Intron
15	58723602	T	C	0.00%	0.00%	ENSG00000166035	100kb Upstream

15	75041350	C	T	0.10%	0.21%	ENSG00000140505	5'UTR
15	79502185	C	T	0.00%	0.00%	ENSG00000235711	100kb Upstream
15	88521571	G	C	0.00%	0.07%	ENSG00000140538	Intron
15	88522371	T	C	0.00%	0.01%	ENSG00000140538	Intron
16	163676	G	A	0.50%	0.45%	ENSG00000103148	Intron
16	209708	T	C	0.00%	0.00%		100kb Downstream
16	222890	C	G	0.00%	0.00%	ENSG00000188536	5'UTR
16	222909	C	T	0.00%	0.00%	ENSG00000188536	5'UTR
16	223690	A	G	0.00%	0.00%	ENSG00000188536	3'UTR
16	223692	A	C	0.00%	0.00%	ENSG00000188536	3'UTR
16	226706	G	C	0.10%	0.01%	ENSG00000206172	5'UTR
16	338078	C	T	0.00%	0.06%	ENSG00000185615	3'UTR
16	3306598	G	C	0.00%	0.00%	ENSG00000103313	5'UTR
16	3306968	G	C	0.10%	0.00%	ENSG00000103313	100kb Upstream
16	16317509	T	G	0.00%	4.25%	ENSG00000091262	100kb Upstream
16	31202799	G	A	0.40%	0.41%	ENSG00000103490	3'UTR
16	31202890	C	A	0.00%	0.02%	ENSG00000103490	3'UTR
16	31202948	C	A	0.00%	0.00%	ENSG00000103490	3'UTR
16	56995795	G	A	0.00%	0.00%	ENSG00000087237	promoter
16	67313931	C	T	0.00%	0.00%	ENSG00000196155	5'UTR
16	67517637	C	T	0.20%	0.07%	ENSG00000159723	5'UTR
16	68771264	G	C	0.20%	0.18%	ENSG00000039068	5'UTR
16	69967004	C	A	0.00%	0.00%	ENSG00000157322	Intron
16	85096736	G	A	0.00%	0.01%	ENSG00000135709	5'UTR
16	85096759	G	A	0.00%	0.00%	ENSG00000135709	5'UTR
17	1303861	C	A	0.00%	0.00%	ENSG00000108953	100kb Upstream
17	3539711	G	T	0.00%	0.00%	ENSG00000040531	promoter
17	4806451	C	T	0.00%	0.00%	ENSG00000108556	100kb Upstream
17	4806452	C	T	0.00%	0.01%	ENSG00000108556	100kb Upstream
17	4806453	G	A	0.00%	0.00%	ENSG00000108556	100kb Upstream
17	7571751	T	G	0.30%	1.20%	ENSG00000129244	3'UTR
17	7906219	T	C	0.00%	0.00%	ENSG00000132518	5'UTR

17	19437134	G	A	0.00%	0.00%	ENSG00000142494	promoter
17	19437208	C	T	0.40%	0.00%	ENSG00000142494	5'UTR
17	30816891	C	G	0.00%	0.00%	ENSG00000176749	3'UTR
17	38721720	G	A	0.00%	0.03%	ENSG00000126353	5'UTR
17	40704915	A	C	0.60%	0.81%	ENSG00000108786	5'UTR
17	41052816	G	A	0.30%	0.75%	ENSG00000131482	promoter
17	41196408	G	A	0.00%	0.00%	ENSG00000108830	3'UTR
17	41196423	A	G	0.00%	0.00%	ENSG00000108830	3'UTR
17	41196894	A	G	0.00%	0.00%	ENSG00000108830	3'UTR
17	41196976	T	C	0.00%	0.00%	ENSG00000108830	3'UTR
17	41197166	C	G	0.00%	0.00%	ENSG00000108830	3'UTR
17	41197636	G	A	0.10%	0.00%	ENSG00000108830	3'UTR
17	42030174	G	T	0.00%	10.26%		3'UTR
17	42078967	C	A	0.00%	0.00%	ENSG00000161653	5'UTR
17	42340295	C	T	0.00%	0.00%	ENSG00000004939	5'UTR
17	42470922	C	T	0.00%	0.00%	ENSG00000005961	100kb Downstream
17	58236873	G	A	0.00%	0.00%	ENSG00000167434	3'UTR
17	61996365	A	G	1.00%	2.46%	ENSG00000259384	100kb Upstream
17	61996673	C	T	0.30%	1.42%	ENSG00000259384	100kb Upstream
17	66508598	G	A	0.00%	0.00%	ENSG00000108946	5'UTR
17	69108654	A	G	0.40%	0.21%	ENSG00000123700	desert
17	73761238	A	G	0.10%	0.05%	ENSG00000108479	5'UTR
17	73839907	G	A	0.00%	0.02%	ENSG00000092929	Intron
17	75316274	G	C	0.00%	0.00%	ENSG00000184640	Intron
18	657684	G	C	0.00%	26.20%	ENSG00000176890	5'UTR
18	55254102	C	G	0.00%	0.00%	ENSG00000066926	100kb Upstream
19	7733792	C	G	0.00%	32.85%	ENSG00000104918	100kb Upstream
19	11199956	G	T	0.90%	0.68%	ENSG00000130164	100kb Upstream
19	11199957	A	G	0.00%	0.00%	ENSG00000130164	100kb Upstream
19	11200007	C	T	0.30%	0.32%	ENSG00000130164	promoter
19	11200018	C	T	0.00%	0.00%	ENSG00000130164	promoter
19	11200036	C	T	0.00%	0.00%	ENSG00000130164	promoter

19	11200063	A	C	0.00%	0.00%	ENSG00000130164	5'UTR
19	11200068	C	T	0.00%	0.00%	ENSG00000130164	5'UTR
19	11200071	C	T	0.00%	0.00%	ENSG00000130164	5'UTR
19	11200072	C	T	0.00%	0.00%	ENSG00000130164	5'UTR
19	11200075	C	A	0.00%	0.00%	ENSG00000130164	5'UTR
19	11200078	C	A	0.00%	0.00%	ENSG00000130164	5'UTR
19	11200082	C	T	0.00%	0.00%	ENSG00000130164	5'UTR
19	11200084	C	G	0.00%	0.00%	ENSG00000130164	5'UTR
19	11200085	C	A	0.00%	0.00%	ENSG00000130164	5'UTR
19	11200086	T	C	0.00%	0.00%	ENSG00000130164	5'UTR
19	11200087	C	T	0.00%	0.00%	ENSG00000130164	5'UTR
19	11200088	C	G	0.00%	0.00%	ENSG00000130164	5'UTR
19	11200089	C	G	0.00%	0.00%	ENSG00000130164	5'UTR
19	11200090	C	T	0.00%	0.00%	ENSG00000130164	5'UTR
19	11200104	C	T	0.00%	0.00%	ENSG00000130164	5'UTR
19	11200201	A	C	0.00%	0.00%	ENSG00000130164	5'UTR
19	11200210	C	A	0.00%	0.00%	ENSG00000130164	5'UTR
19	11200211	A	G	0.00%	0.00%	ENSG00000130164	5'UTR
19	11200219	C	T	0.00%	0.00%	ENSG00000130164	5'UTR
19	11242034	G	A	0.00%	0.00%	ENSG00000161888	3'UTR
19	12992335	C	T	0.00%	0.00%	ENSG00000105612	promoter
19	12998077	A	G	0.00%	0.00%	ENSG00000105610	100kb Upstream
19	13049193	C	A	0.00%	0.04%	ENSG00000179218	100kb Upstream
19	13049217	C	T	0.00%	0.01%	ENSG00000179218	100kb Upstream
19	13049374	G	C	0.00%	0.00%	ENSG00000179218	promoter
19	18496124	C	T	0.00%	0.00%	ENSG00000130513	100kb Upstream
19	35773327	C	T	0.20%	0.26%	ENSG00000105697	5'UTR
19	35773452	G	T	0.00%	0.00%	ENSG00000105697	5'UTR
19	35773455	G	A	0.00%	0.00%	ENSG00000105697	5'UTR
19	39138351	C	T	0.00%	0.00%	ENSG00000130402	5'UTR
19	41349496	G	T	0.00%	1.17%		3'UTR
19	41349499	T	C	0.00%	1.14%		3'UTR

19	41357343	T	C	0.00%	56.67%		100kb Upstream
19	41930735	T	A	0.00%	0.00%	ENSG00000177191	3'UTR
19	49468349	C	A	0.00%	0.00%	ENSG00000087086	100kb Upstream
19	49468574	C	T	0.00%	0.00%	ENSG00000087086	promoter
19	49468578	C	G	0.00%	0.00%	ENSG00000087086	5'UTR
19	49468580	C	T	0.00%	0.00%	ENSG00000087086	5'UTR
19	49468582	C	T	0.00%	0.00%	ENSG00000087086	5'UTR
19	49468586	T	G	0.00%	0.00%	ENSG00000087086	5'UTR
19	49468588	T	C	0.00%	0.00%	ENSG00000087086	5'UTR
19	49468593	C	G	0.00%	0.00%	ENSG00000087086	5'UTR
19	49468596	G	C	0.00%	0.00%	ENSG00000087086	5'UTR
19	49468597	C	A	0.00%	0.00%	ENSG00000087086	5'UTR
19	49468598	T	C	0.00%	0.00%	ENSG00000087086	5'UTR
19	49468600	C	G	0.00%	0.00%	ENSG00000087086	5'UTR
19	49468601	A	G	0.00%	0.00%	ENSG00000087086	5'UTR
19	49468603	C	A	0.00%	0.00%	ENSG00000087086	5'UTR
19	49468604	A	G	0.00%	0.00%	ENSG00000087086	5'UTR
19	49468605	G	C	0.00%	0.00%	ENSG00000087086	5'UTR
19	49468607	G	A	0.00%	0.00%	ENSG00000087086	5'UTR
19	49468610	T	G	0.00%	0.00%	ENSG00000087086	5'UTR
19	49468611	G	A	0.00%	0.00%	ENSG00000087086	5'UTR
19	49468613	A	C	0.00%	0.00%	ENSG00000087086	5'UTR
19	49468614	C	A	0.00%	0.00%	ENSG00000087086	5'UTR
19	49468615	G	C	0.00%	0.00%	ENSG00000087086	5'UTR
19	49468620	A	T	0.00%	0.00%	ENSG00000087086	5'UTR
19	49468654	C	T	0.00%	0.00%	ENSG00000087086	5'UTR
19	52196527	G	T	0.00%	0.00%	ENSG00000105509	Intron
19	52264212	A	G	0.00%	0.00%		100kb Upstream
19	55327927	G	T	0.00%	0.00%	ENSG00000167633	5'UTR
20	23030442	G	T	0.00%	0.00%	ENSG00000178726	100kb Upstream
20	32891109	G	A	0.20%	1.00%	ENSG00000101444	5'UTR
20	33578226	T	C	0.00%	0.00%	ENSG00000078814	Intron

20	33759639	T	G	0.00%	0.00%	ENSG00000101000	100kb Upstream
20	42984252	C	G	0.00%	0.00%	ENSG00000101076	100kb Upstream
20	42984263	G	A	0.00%	0.00%	ENSG00000101076	100kb Upstream
20	42984275	C	T	0.00%	0.00%	ENSG00000101076	100kb Upstream
20	42984308	A	G	0.00%	0.00%	ENSG00000101076	100kb Upstream
20	43058471	T	A	1.00%	1.05%	ENSG00000168746	3'UTR
20	48552878	C	A	0.00%	0.00%	ENSG00000124226	promoter
20	48552880	C	A	0.00%	0.02%	ENSG00000124226	promoter
20	48552903	C	T	0.00%	0.00%	ENSG00000124226	promoter
20	48552935	A	C	0.00%	0.00%	ENSG00000124226	5'UTR
20	57875742	G	A	0.00%	0.00%	ENSG00000124205	5'UTR
20	57875848	C	A	0.00%	0.02%	ENSG00000124205	5'UTR
21	27543202	G	T	0.00%	0.00%	ENSG00000142192	5'UTR
21	27543453	G	C	0.00%	0.01%	ENSG00000142192	promoter
21	27543618	C	T	0.00%	0.09%	ENSG00000142192	100kb Upstream
21	27546913	G	A	0.00%	0.00%	ENSG00000142192	100kb Upstream
21	34668713	C	T	0.00%	0.00%	ENSG00000142166	3'UTR
21	43639008	C	T	0.10%	0.17%	ENSG00000160179	5'UTR
22	19710932	C	G	0.00%	0.00%	ENSG00000184702	100kb Downstream
22	19743577	C	T	0.00%	0.00%	ENSG00000184058	100kb Upstream
22	19743734	A	C	0.10%	0.01%	ENSG00000184058	100kb Upstream
22	19747127	C	T	0.30%	0.05%	ENSG00000184058	5'UTR
22	24176448	C	T	0.00%	0.00%	ENSG00000099958	3'UTR
22	24322259	T	C	0.00%	0.00%	ENSG00000099977	5'UTR
22	24384525	G	C	0.00%	0.00%	ENSG00000184674	100kb Upstream
22	24384706	C	T	0.10%	0.38%	ENSG00000184674	100kb Upstream
22	24384944	G	A	0.00%	0.33%	ENSG00000184674	100kb Upstream
22	30642689	G	T	0.00%	0.08%	ENSG00000128342	5'UTR
22	35777153	C	T	0.10%	0.12%	ENSG00000100292	5'UTR
22	37415491	C	G	0.10%	0.21%		promoter
22	40742513	T	C	0.00%	0.00%	ENSG00000239900	promoter
23	591567	C	A	0.00%	0.00%	ENSG00000185960	5'UTR

23	22266300	A	G	0.00%	0.00%	ENSG00000175809	3'UTR
23	33229482	A	T	0.00%	0.00%	ENSG00000185448	5'UTR
23	37639261	A	C	0.00%	0.00%	ENSG00000165168	promoter
23	37639263	T	C	0.00%	0.00%	ENSG00000165168	promoter
23	37639265	C	T	0.00%	0.00%	ENSG00000165168	promoter
23	37639266	C	T	0.00%	0.00%	ENSG00000165168	promoter
23	38211583	A	G	0.20%	0.30%	ENSG00000036473	100kb Upstream
23	43832684	T	C	0.00%	0.02%	ENSG00000124479	5'UTR
23	49028159	C	A	0.60%	37.09%	ENSG00000102007	promoter
23	49106916	T	C	0.00%	0.00%	ENSG00000049768	3'UTR
23	49106918	T	C	0.00%	0.00%	ENSG00000049768	3'UTR
23	49114968	C	A	0.00%	0.00%	ENSG00000049768	5'UTR
23	49122081	G	C	0.60%	0.30%	ENSG00000049768	100kb Upstream
23	49779217	C	G	0.00%	0.00%	ENSG00000171365	Intron
23	55057392	G	A	0.00%	0.03%	ENSG00000158578	5'UTR
23	55057616	G	C	0.30%	0.54%	ENSG00000158578	100kb Upstream
23	70327277	T	C	0.00%	0.00%	ENSG00000204165	3'UTR
23	70443028	T	G	0.00%	0.00%	ENSG00000169562	promoter
23	70443030	G	C	0.00%	0.00%	ENSG00000169562	promoter
23	70443098	C	T	0.00%	0.00%	ENSG00000169562	5'UTR
23	70443184	G	A	0.00%	0.00%	ENSG00000169562	5'UTR
23	70838053	C	T	0.00%	25.13%	ENSG00000186810	5'UTR
23	79277759	C	G	0.00%	0.34%	ENSG00000122145	5'UTR
23	100641043	A	C	0.00%	0.00%	ENSG00000010671	5'UTR
23	100641211	T	C	0.00%	0.00%	ENSG00000010671	promoter
23	100662920	C	T	0.30%	1.06%		5'UTR
23	103031892	C	T	0.70%	0.17%	ENSG00000123560	5'UTR
23	119603166	C	T	0.00%	0.00%	ENSG00000005893	5'UTR
23	119604077	T	G	0.00%	0.00%	ENSG00000005893	100kb Upstream
23	135633050	T	G	0.00%	0.00%	ENSG00000102243	Intron
23	138612868	G	T	0.00%	0.00%	ENSG00000101981	promoter
23	138612870	A	G	0.00%	0.00%	ENSG00000101981	promoter

23	138612871	C	G	0.00%	0.00%	ENSG00000101981	promoter
23	138612873	T	G	0.00%	0.00%	ENSG00000101981	promoter
23	138612874	T	A	0.00%	0.00%	ENSG00000101981	promoter
23	138612875	G	C	0.00%	0.00%	ENSG00000101981	promoter
23	138612888	G	C	0.00%	0.00%	ENSG00000101981	promoter
23	138612889	A	T	0.00%	0.00%	ENSG00000101981	promoter
23	138612899	T	A	0.00%	0.00%	ENSG00000101981	promoter
23	138612900	T	C	0.00%	0.00%	ENSG00000101981	promoter
23	138612901	T	C	0.00%	0.00%	ENSG00000101981	promoter
23	138612902	C	G	0.00%	0.00%	ENSG00000101981	promoter
23	138612905	A	G	0.00%	0.00%	ENSG00000101981	5'UTR
23	138612906	A	G	0.00%	0.00%	ENSG00000101981	5'UTR
23	138612918	A	G	0.00%	0.00%	ENSG00000101981	5'UTR
23	138645386	A	G	0.00%	0.00%	ENSG00000101981	3'UTR
23	138645597	A	G	0.00%	0.00%	ENSG00000101981	3'UTR
23	145075803	C	G	0.50%	0.74%	ENSG00000221870	desert
23	146353878	A	G	0.00%	0.00%	ENSG00000102081	desert
23	146353920	C	T	0.00%	0.00%	ENSG00000102081	desert
23	146993614	G	C	0.00%	0.00%	ENSG00000102081	5'UTR
23	148075199	T	C	0.20%	0.35%	ENSG00000155966	3'UTR
23	148076067	C	T	0.00%	0.01%	ENSG00000155966	3'UTR
23	150573535	A	G	0.00%	0.00%	ENSG00000160131	3'UTR
23	153991098	C	G	0.00%	0.21%	ENSG00000130826	5'UTR
23	154250831	T	C	0.00%	0.00%		5'UTR
23	154250938	C	T	0.00%	0.00%		5'UTR
23	154251045	G	A	0.00%	0.00%		promoter
23	154251081	T	C	0.00%	0.00%		100kb Upstream
23	154251083	A	C	0.00%	0.00%		100kb Upstream
23	154251686	T	C	0.00%	0.00%		100kb Upstream
24	2655718	C	T	0.00%	0.00%	ENSG00000184895	5'UTR
24	2655773	C	G	0.00%	0.00%	ENSG00000184895	promoter

Supplementary Table 4. Available GTEx variant scores.

GTEx Group	Variants	CADD	LINSIGHT	EIGEN	DeepSEA	GWAVA	CATO
Largest Effect Size	552	517	542	506	516	518	97
Most Likely Causal	429	398	421	391	398	397	90
Best p-value	304	295	285	282	295	295	67
